# Modeling the acceptability of BCIs for motor rehabilitation after stroke: A large scale study on the general public

**DOI:** 10.3389/fnrgo.2022.1082901

**Published:** 2023-02-01

**Authors:** Elise Grevet, Killyam Forge, Sebastien Tadiello, Margaux Izac, Franck Amadieu, Lionel Brunel, Léa Pillette, Jacques Py, David Gasq, Camille Jeunet-Kelway

**Affiliations:** ^1^CNRS, EPHE, INCIA, UMR5287, Université de Bordeaux, Bordeaux, France; ^2^CLLE, Université de Toulouse, CNRS, Toulouse, France; ^3^Université Paul Valéry Montpellier 3, EPSYLON EA 4556, Montpellier, France; ^4^ToNIC, Université de Toulouse, INSERM, Toulouse, France; ^5^Centre Hospitalier Universitaire Toulouse, Toulouse, France

**Keywords:** brain-computer interface (BCI), neurofeedback (NF), acceptability, acceptance, stroke, motor rehabilitation, model, questionnaire

## Abstract

**Introduction:**

Strokes leave around 40% of survivors dependent in their activities of daily living, notably due to severe motor disabilities. Brain-computer interfaces (BCIs) have been shown to be efficiency for improving motor recovery after stroke, but this efficiency is still far from the level required to achieve the clinical breakthrough expected by both clinicians and patients. While technical levers of improvement have been identified (e.g., sensors and signal processing), fully optimized BCIs are pointless if patients and clinicians cannot or do not want to use them. We hypothesize that improving BCI acceptability will reduce patients' anxiety levels, while increasing their motivation and engagement in the procedure, thereby favoring learning, ultimately, and motor recovery. In other terms, acceptability could be used as a lever to improve BCI efficiency. Yet, studies on BCI based on acceptability/acceptance literature are missing. Thus, our goal was to model BCI acceptability in the context of motor rehabilitation after stroke, and to identify its determinants.

**Methods:**

The main outcomes of this paper are the following: i) we designed the first model of acceptability of BCIs for motor rehabilitation after stroke, ii) we created a questionnaire to assess acceptability based on that model and distributed it on a sample representative of the general public in France (*N* = 753, this high response rate strengthens the reliability of our results), iii) we validated the structure of this model and iv) quantified the impact of the different factors on this population.

**Results:**

Results show that BCIs are associated with high levels of acceptability in the context of motor rehabilitation after stroke and that the intention to use them in that context is mainly driven by the *perceived usefulness* of the system. In addition, providing people with clear information regarding BCI functioning and scientific relevance had a positive influence on acceptability factors and *behavioral intention*.

**Discussion:**

With this paper we propose a basis (model) and a methodology that could be adapted in the future in order to study and compare the results obtained with: i) different stakeholders, i.e., patients and caregivers; ii) different populations of different cultures around the world; and iii) different targets, i.e., other clinical and non-clinical BCI applications.

## 1. Introduction

Brain-Computer Interfaces (BCIs) are technologies that enable users to control applications such as video games (Kerous et al., 2018) or wheelchairs (Li et al., 2013), solely through their brain activity. Beyond these control applications, BCIs can be used for neurofeedback (NF) training with the objective of learning how to modulate our own cerebral activity, not in order to control something, but to improve or restore cognitive or motor skills. BCI-based post-stroke motor rehabilitations are in this second category and have demonstrated their efficacy to improve patients' motor and cognitive abilities (Cervera et al., 2018; Bai et al., 2020; Nojima et al., 2022). In the coming years, they are expected to substantially improve post-stroke subjects' quality of life (Nojima et al., 2022).

In classical motor rehabilitation, when subjects have no residual movement, i.e., when they cannot move their affected limb at all, physical practice is impossible and both subjects and therapists must rely on mental practice alone. Here, in mental practice, we include motor imagery (MI) as well as attempted movements. In concrete terms, therapists usually ask the subjects to perform MI or to try to move their arm (attempted movements), and simultaneously stimulate the limb by mobilizing it or, for instance, by using functional electrical stimulation (FES-which consists in stimulating peripheral motor nerves in order to artificially generate movements). While associated to encouraging results (Sharma et al., 2006), the difficulty encountered when trying to demonstrate the efficiency of this procedure might be related to the impossibility to assess the patients' compliance when they are asked to perform MI tasks (Sharma et al., 2006). In addition, we believe pure mental practice-based rehabilitation procedures present two main limitations. The first one is due to the impossibility of the therapist to know when, exactly, the patient imagines moving or tries to move. Therefore, the feedback patients are provided with will most likely not be synchronized with their MI or movement attempts. A second limitation concerns the constant reminder that the patient gets when the therapist asks them to move their arm and that they are unable to do so. Post-stroke subjects experiencing high anxiety levels (Burton et al., 2013), this method might also have detrimental psychological effects, potentially resulting in the patient disengaging from the rehabilitation procedure, and in the therapy being less efficient.

In this context, BCIs are very relevant as they enable the detection of MI/attempted movements of the impaired limb, which are underlain by modulations of the so-called sensori-motor rhythms (SMRs)—as defined in the BCI field by a large band covering mu (μ) and beta (β) rhythms (8–30 Hz) (Pfurtscheller et al., 2000)—, and provide the patient with a synchronized NF, for instance using FES that triggers an arm muscle contraction, or visual feedback [movement of a virtual hand on a screen (Pichiorri et al., 2015)]. Such a NF training enables the participants to train to voluntarily self-regulate their SMRs in a closed loop process, which should favor synaptic plasticity and motor recovery (Jeunet et al., 2019).

While this is encouraging, BCI efficiency is still far from the level required to achieve the clinical breakthrough expected by both clinicians and patients. Thus, BCIs remain barely used in clinical practice, outside laboratories (Kübler et al., 2014). BCI efficiency is known to be modulated by several factors. Many researchers are working on improving this efficiency either from a “technical” point of view (e.g., signal processing Lotte et al., 2018), or—less often—from the human learning standpoint (Pillette et al., 2020; Roc et al., 2021). This is an important step forward: reaching high efficiency is a necessary condition for BCI adoption. Nonetheless, it might not be sufficient for those technologies to be actually used in a clinical setting: fully optimized BCIs (in terms of sensors, signal processing, and training procedures) are pointless if patients and clinicians are not able or do not want to use them, i.e., if BCIs are not accepted (Blain-Moraes et al., 2012). For instance, misconceptions that patients and their entourage have regarding BCIs may have a detrimental effect on the acceptance of these technologies. BCI acceptance could also be altered by the fact that most stroke patients experience depression, and therefore high anxiety levels (Burton et al., 2013) that have a detrimental effect on BCI acceptance and learning (Jeunet et al., 2016). Thus, BCI acceptance is likely to have a major impact on the patients' learning processes and therefore on the efficiency of BCI-based stroke rehabilitation procedures. We hypothesize that identifying acceptability and acceptance factors will help us overcome these misconceptions and personalize the procedures, which will in turn result in reduced anxiety, and increased motivation and engagement levels for the patients. This should favor their learning and, ultimately, motor recovery. In other words, we expect that improving the acceptance levels of BCIs will result in an increased efficiency of these technologies and therefore contribute to their democratization.

Thus, it is crucial, when designing stroke rehabilitation procedures, to consider technology acceptance as a lever to optimize BCI efficiency—in terms of motor recovery. Yet, BCI acceptance remains an aspect that has been little studied to date. To the best of our knowledge, only (Morone et al., 2015) assessed the relevance of a BCI-based stroke rehabilitation procedure of the upper limb using acceptability and usability measures as primary criteria (pilot study, *N* = 8 patients). The acceptability and usability were measured in terms of mood, motivation, satisfaction, and perceived workload. Indeed, in the BCI field, acceptability is mostly assessed as an attribute of the user's satisfaction, itself being a dimension of user experience (Kübler et al., 2014; Nijboer, 2015). Morone et al. (2015) concludes that the BCI training was “accepted with a good compliance/adherence.” The same conclusion was drawn in the context of a BCI dedicated to a gamified cognitive training for the elderly (Lee et al., 2013) as well as in different studies dedicated to the acceptance of BCIs by Amyotrophic Lateral Sclerosis (ALS) patients (Huggins et al., 2011; Blain-Moraes et al., 2012; Nijboer, 2015), which is the clinical condition for which acceptance has been the most investigated (Nijboer, 2015). Using a focus group approach, Blain-Moraes et al. (2012) have shown that both personal and relational factors impacted BCI acceptance. The personal factors included physical (pain, discomfort), physiological (fatigue, endurance) and psychological (anxiety, attitude toward the technology) concerns, and the relational factors included corporeal (electrode type), technological (relationship between BCI and other type of software and hardware) and social (appearance, training and support personnel) factors. The relational factors had a stronger impact than the personal ones. In the same line, Huggins et al. (2011, 2015) led qualitative studies to assess the influence that different factors (physical interface, setup and training, acceptable performance, task and feature priorities) had on BCI acceptance in ALS patients and patients who had undergone a spinal cord injury. The functions provided by the BCI were rated as the most important feature, together with the ease of use of the system and the availability of a stand-by mode. Finally, Geronimo et al. (2015) have shown that behavioral impairments such as apathy and mental rigidity had a negative impact on ALS patients' BCI usage behavior. Furthermore, the fact that they performed a pilot study during which patients appeared to have a low perceived control over the system altered the perceived usefulness of the BCI. This latter study is the only one in the field of BCIs that assessed acceptance in terms of usage behavior and perceived usefulness. These are concepts from the field of psychology and ergonomics, which we draw on in the next paragraph (Kaleshtari et al., 2016). As claimed by Kaleshtari et al. (2016), who designed the first Model of Rehabilitation Technology Acceptance and Usability (RTAU), in order to be effective, rehabilitation technologies have to be used and therefore accepted by the patients and their families. According to Kaleshtari et al. (2016), this acceptance depends both on personal features, technology features, and social influence. The domain-specific literature indeed suggests that BCI acceptability and acceptance seem to rely on “subjective technical confidence and positive attitudes toward the use of technologies” (Morone et al., 2015). The EEG cap characteristics (gel, montage, and time to set up) seem important both for patients and caregivers (Morone et al., 2015; Nijboer, 2015). Generally speaking, the simpler the better for them (Huggins et al., 2011). Nonetheless, the EEG technology used and the reliability/discomfort trade-off, together with all the BCI-related characteristics, need to be thought of in light of the characteristics and requirements of the application (e.g., level of reliability required) as well as in light of the profile of the patient. This is why BCI-based stroke rehabilitation procedures should be carefully adapted to the training context of each patient. In this spirit, Kübler et al. (2014) proposed an inspiring approach, suggesting to “shift from focusing on single aspects, such as accuracy and information transfer rate, to a more holistic user experience” using User-Centered Design. User-Centered Design has since then been shown to contribute to the acceptability and usability of a BCI-based stroke rehabilitation procedure (Morone et al., 2015) and, more broadly, improved user experience has been suggested to enhance user acceptance and increase the performance of BCI systems (Gürkök et al., 2011).

The concepts of acceptability and acceptance were introduced in order to understand what led users to adopt or not a new system (Alexandre et al., 2018). The adoption of a technology refers to a use that is maintained over time, i.e., without abandonment. In concrete terms, acceptability measure is an evaluation of the user's behavioral intention (BI) i.e., their intention to use the studied technology. The main determinants of BI are perceived usefulness (PU) and perceived ease of use (PEOU). PU is the personal feeling about utility of the system, and PEOU the degree of belief to which using the system will require little or no effort. Acceptability and acceptance differ by the moment they are measured at: acceptability concerns the user's standpoint before any interaction with the system, while acceptance comes after at least one first use.

To the best of our knowledge, there is no model of BCI acceptability yet. Thus, our goals were to (i) create a theoretical model of acceptability—based on the literature—including the factors influencing BI toward BCIs, especially in the context or motor rehabilitation after stroke; (ii) implement a questionnaire from this model to assess BCI acceptability in general public; (iii) validate the structure of our model and questionnaire; and (iv) quantify the impact of the different factors included in the model on acceptability.

With this study we targeted the general public. This enabled us to collect the opinions and attitudes of a large sample of persons, representative of the adult population in France, and thereby to capture an estimation of BCI acceptability in the overall population that we will, in the future, compare with the one of patients and clinicians. Targeting the general public seems particularly relevant in this case for two reasons. First, due to the high prevalence of stroke that is one of the leading causes of disability in adults in France (Accident Vasculaire cérébral, 2019) and results in many of us being concerned, more or less directly, by this pathology and associated rehabilitation techniques. Second, due to the fact that the opinion and attitude of their close relatives will influence the patients' acceptability levels (Venkatesh et al., 2003).

In this paper, we first explain our methodology to achieve these objectives in Section 2: (i) the design of the model, (ii) the implementation of the associated questionnaire, (iii) the validation of these model and questionnaire, and (iv) the quantification of the influence of the different factors included in the model on BCI acceptability. Then, we present the results of this methodology in Section 3, which are based on data collected on a sample representative of the population of France (*N* = 753). We finish with a discussion about limitations and benefits of our research.

## 2. Materials and methods

### 2.1. Design of the acceptability model

#### 2.1.1. Review of the literature

To build our model, we reviewed the literature and selected the models that seemed the most relevant for BCIs.

In the literature, several models dedicated to the acceptability and acceptance of technologies have been depicted, most of them can be adapted depending on the focus (i.e., acceptability or acceptance). Their objective is usually to explain, or even predict, the BI of the user. These models differ from each other in the factors they include and that influence acceptance/acceptability. The most recent and main models are the technology acceptance model (TAM) (Davis, 1989), of which there are a second (Venkatesh and Davis, 2000) and third (Venkatesh and Bala, 2008) versions, as well as the unified theory of acceptance and use of technology (UTAUT) (Venkatesh et al., 2003), of which there is a second version (Venkatesh et al., 2012). For more information about other existing acceptability models, their evolution is summarized in a recent review (Pillette et al., 2022).

#### 2.1.2. Methodology to build our model

We have chosen to work with the most advanced versions of the evoked models: TAM3 (Venkatesh and Bala, 2008) and UTAUT2 (Venkatesh et al., 2012), in addition to a less widespread model, the components of user experience (CUE) model (Thüring and Mahlke, 2007), because we wanted up-to-date models that were adapted to our context and as exhaustive as possible. We propose to present these latter and the details of why we chose them in Table 1.

**Table 1:** Acceptability and acceptance models used to build our model for BCI-based post-stroke motor rehabilitation.

**Technology acceptance model 3 (TAM3)-Venkatesh and Bala (** **2008** **)**	
Theory	TAM3 is the third version of TAM (Davis, 1989). In TAM3, the main concepts are **perceived usefulness (PU)** and **perceived ease of use (PEOU)**. PU is the degree to which a person believes that using a system will improve their performances; and PEOU, the degree to which using a system will be effortless. These factors are determined by the characteristics of the technology and influence the behavioral intention (BI). TAM3 also includes the *social influence* process which is constituted of **subjective norm** and **image**. Subjective norm is the subjective perception of individuals about what people who are important to them will think concerning the usage of a technology. According to the authors, the presence or absence of important effects of subjective norm on the BI is moderated by **voluntariness**, i.e., the voluntary or forced aspect of use, and by **previous experience**. The subjective norm can have a stronger impact when the use is constrained (Venkatesh and Davis, 2000). Concerning image, it can be defined as the degree of users' perception on whether or not the use of a technology will improve their status within the social group to which they belong (Moore and Benbasat, 1991).
	Another category is the *extrinsic motivational processes*, sometimes referred to the instrumental cognitive process (i.e., **relevance of the technology**, **perceived quality**, and **result demonstrability**). These factors allow users to form a judgement on what the use of technology can bring them given their needs to achieve their goals.
	In TAM3, the novelty in comparison to TAM2 (Davis et al., 1992) is the addition of determinants of PEOU. PEOU is determined by *intrinsic motivational processes* also called the anchored beliefs, i.e., playfulness of the interaction, perception of external control, self-efficacy and computer anxiety. **Computer playfulness** is the enjoyment when using the studied technology. **Perception of external control** are the “individuals' control beliefs regarding the availability of organizational resources and support structure to facilitate the use of a system” (Venkatesh and Bala, 2008). **Self-efficacy** can be defined as the “individuals' control beliefs regarding his or her personal ability to use a system” (Venkatesh and Bala, 2008). Finally, **computer anxiety** is an emotion that corresponds to the more or less conscious expectation of a danger or a problem to come associated with the use of the system. PEOU is in addition determined by **adjustments** factors that include enjoyment of use and objective usability. These adjustments are made by the user during the interaction, which appears only in the dimension of acceptance and no longer in acceptability like the other factors.
Justifications	A large majority of studies dedicated to the assessment of the acceptance of new technologies have used the first version of TAM (studies between 2001 and 2018 in the review of Alturas, 2021, and between 2006 and 2015 in Rad et al., 2018). This can be explained in part by the fact that this version is made up of fewer factors than TAM2 and TAM3, in consequence its implementation and test in a given research context can be easier. In our case, we preferred to use the most advanced version of TAM. We privileged the completeness of the model instead of the simplicity of implementation as we are in an exploratory stage of research on BCIs acceptability (the factors deletion will be addressed in a near future, in accordance with the results of our study and of other future papers).
**Unified theory of acceptance and use of technology 2 (UTAUT2)-Venkatesh et al. (** **2012** **)**	
Theory	UTAUT2 is the second version of UTAUT (Venkatesh et al., 2003) which unifies the concepts of 8 models of acceptability. UTAUT is composed of four determinants—social influence, facilitating conditions, performance expectations and effort expectations—that influence the BI. It also includes four moderators: gender, age, experience and voluntary aspect of use. **Performance expectation** and **effort expectation** factors correspond to the PU and PEOU factors of TAM, respectively. **Facilitating conditions** are the material, organizational and/or human conditions facilitating the use of a technology (Février, 2011). In comparison to the first version, UTAUT2 adds three determinants of BI, namely hedonic motivation, price value and habit. **Hedonic motivation** is the pleasure provided by the use of a technology, this factor is close to computer playfulness factor of TAM3. **Price value** refers to the trade-off made by consumers between the perceived advantages of the technology and its cost. On the other hand, the authors have chosen to delete one of the moderators: the voluntariness no longer appears in UTAUT2.
Justifications	UTAUT has the advantage of being created from previous reliable models, which makes it a robust reference. Moreover, in order to test the quality of UTAUT, its designers had created a questionnaire with items from the eight models. In their study, the questionnaire was circulated in four different organizations among employees being introduced to a new technology in their workplace. It appeared that UTAUT determinants achieved to predict 70% (adjusted R^2^) of the BI. This score was the most accurate among the previous models (Venkatesh et al., 2003; Koul and Eydgahi, 2017). Its extension (UTAUT2) is of greater relevance to us since it was designed to be more suitable for consumer technologies rather than for an organizational setting (Rondan-Cataluña et al., 2015), which fits better with the use cases of BCIs.
**Components of user experience (CUE)-Thüring and Mahlke (** **2007** **)**	
Theory	In order to widen the field of research in human-computer interactions, authors (Dillon, 2001; Hassenzahl, 2001, 2003, 2004; Mahlke, 2008) have proposed an approach which considers a system not only with regard to its properties and its functional benefits, but also to the experience of use (emotional reaction, pleasure of use, etc.) (Février, 2011). CUE is in this line, it highlights the interaction of 3 components of user experience: (i) the perception of the instrumental qualities of the system (referring to the PU and PEOU), (ii) the perception of its non-instrumental qualities (aesthetics, motivational aspects and values conveyed), and (iii) the user's emotional reactions (subjective feelings, motor and behavioral expressions, physiological reactions, and cognitive evaluation) when interacting with the technology. These 3 dimensions lead to the formation of judgments and behaviors toward the use of a given technology.
Justifications	What particularly interested us in CUE was the higher level of consideration—in comparison to TAM3 and UTAUT2—of the attractiveness of the technology (aesthetics, motivational aspects and conveyed values) (Barcenilla and Bastien, 2009). This is indeed a point to be studied in the case of a technology such as an EEG-based BCI for which the visual aspect of the interface may seem cumbersome for users, especially novices.

Using the models presented in Table 1, our work was to identify their factors that seemed relevant in the context of BCI-based post-stroke motor rehabilitation, while reflecting on missing determinants that are however useful to assess with regard to the literature on BCIs.

### 2.2. Creation and distribution of the questionnaire

As we explained in introduction, from our acceptability model, we developed a questionnaire to identify and weight the factors influencing the acceptability of BCI-based post-stroke motor rehabilitation within the general population. The choice to rely on the questionnaire method to test our model is explained by several aspects: (i) It is the classic method used in acceptability or acceptance assessment (Davis, 1989; Venkatesh et al., 2003). (ii) It was necessary to be able to collect a large amount of data, on a sample representative of the adult population in France, and questionnaires are particularly adapted to these expectations (Vilatte, 2007). (iii) In addition, questionnaires offer good external validity (Ghiglione and Matalon, 1998), which makes it possible to generalize the data, the information being more uniform than interviews results.

#### 2.2.1. Creating the questions

**Figure 1:**
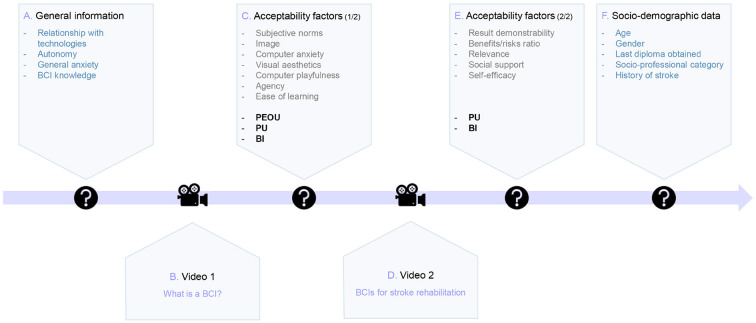
Schematic representation of the structure of the questionnaire: **(A)** Assessment of the respondents' traits and general knowledge about BCIs; **(B)** Presentation of the first video that aimed at providing basic information regarding BCIs (functioning, installation, etc.); **(C)** Items related to a subset of acceptability factors (1/2); **(D)** Presentation of the second video during which the application of BCIs for post-stroke motor rehabilitation was introduced. **(E)** Items related to a subset of acceptability factors (2/2); **(F)** Collection of socio-demographic data. The two subsets of acceptability factors were divided depending on the need for respondents to have knowledge about how BCIs could be used for stroke rehabilitation.

To determine the wording of the questions, we adapted those of the questionnaires of the existing models already translated into French. The questionnaire was created on the Qualtrics tool, it was fully anonymous, and therefore not subject to the general data protection regulation (GDPR). It took approximately 15 min to be completed and consisted of four parts:

- To start, participants were provided with all the information they should know about the research project: objectives of the questionnaire, researchers involved, benefits and possible risks of filling the questionnaire, rights (e.g., anonymity preserved), methodology used and estimated completion time. Finally, the participants were asked if they consented to participate.
- Following these details, the experience of the participant with BCIs was assesed as it could have an influence on some of the predictive factors of BI.
- The third part was devoted to the evaluation of the influence of the factors of our model. Each of them was evaluated by up to three or five questions. The score of a factor was thus the average of the scores of these different questions. The scale used to measure each of the quantitative factors was a visual analog scale (VAS) from 0 to 10 (“strongly disagree” to “strongly agree”). When a question was negative (e.g., “*I think learning to use a brain-computer interface would be too time consuming”*), we inverted its score. To measure the categorical factors (*computer self-efficacy* and *social support*), we used checkbox questions.
  In this part, we also introduced two explanatory videos edited by ourselves: one explaining the operation of BCIs in general (video 1) and the second more specific to BCI-based stroke rehabilitation procedures (video 2). The rationale of providing these explanatory videos is presented in the next paragraph.
  We provide in Figure 1 more details on the organization of our questionnaire. The questions were organized in blocks (a block is made up of 2 or more factors). To avoid any potential order effect, i.e., that the previous questions would guide the following answers, the order of presentation of the questions was randomized within each block.
- The last part concerned the socio-demographic characteristics of the respondents (age, gender, last diploma obtained, socio-professional category, if they have had a stroke and are currently hospitalized for it, or if they have people in their close circle who have experienced it and their involvement in the rehabilitation of these relatives). Subjects were not obligated to position themselves, they had the possibility to choose the option “I do not wish to answer.”

Concerning the factors, we evaluated different factors before and after the second video (Figure 1). PU and BI were measured twice (before video 2: PU1/BI1; after video 2: PU2/BI2), the questions were the same for the two moments. Our aim was to observe if the respondents' scores for the two measures were impacted by information given in the video. The factors before video 2 did not require to have plenty of information on BCIs to answer, i.e., the respondents needed to understand what BCIs are but remained novices on their use in post-stroke rehabilitation. On the other hand, for the factors following video 2, a more detailed vision of these new rehabilitation procedures was needed (factors were *result demonstrability, benefits/risk ratio* and *relevance*). Questionnaire (French and English versions) is available in Supplementary material 1.

#### 2.2.2. Calculation of the statistical power

Our initial target was to have at least 10 respondents per item (i.e., question) on the factors influencing acceptability in order to be able to perform reliable analyzes (Kline, 2015). As we had 62 items, this gave us a sample size of *N* = 620 to respect this prerequisite.

#### 2.2.3. Distribution of the questionnaire

The distribution of the questionnaire was done by the company Panelabs (https://fr.panelabs.com/) to ensure that the sample of respondents was representative of the adult population in France in terms of age, gender, place of residence and socio-professional category. We had a single exclusion criterion: minors could not participate. The experimental protocol was carried out in accordance with the Declaration of Helsinki and was approved by Institutional Review Board of Toulouse Federal University (N°2019-140). We fixed with Panelabs a sample of *N* = 665 minimum in order to have a few more respondents than our aim (i.e., *N* = 620), in case of invalid responses.

### 2.3. Validation of the structures of the model and questionnaire

The assessment of the validity of the structure of our model and questionnaire was performed following two steps. First, we measured the “within-factor” consistency, i.e., the internal coherence between the different items of the questionnaire that measured the same factor. Second, we assessed the “between-factor” consistency, i.e., the validity of the structure of our model.

#### 2.3.1. Coherence of the factors: Cronbach's alpha

The Cronbach's alpha coefficient allowed us to calculate the internal consistency of each factor. Concretely, this metric estimates the extent to which the items that are meant to measure one same factor are associated with coherent scores. There is no fixed rule on the minimal value of the coefficient for the internal consistency of the factor to be considered satisfactory. Nevertheless, the value 0.7 comes up very often in the literature (Nunnally, 1994; Bland and Altman, 1997; DeVellis and Thorpe, 2021). It is also indicated that a coefficient too close to 1 is to be taken with precaution, this high value may be due to redundancy in the question statements (Tavakol and Dennick, 2011). In other words, the items would be too similar one from the other, and do not bring additional information.

#### 2.3.2. Structure of the model and questionnaire: Confirmatory factor analysis

Confirmatory factor analysis (CFA) is a validation test, which also aims to verify the internal consistency of the questionnaire and check whether the model we propose fits correctly with the data collected. Several indicators are used to interpret the CFA (Gallagher and Brown, 2013): **(i)** The chi-square (*X*^2^) test which has the null hypothesis that the model fits perfectly. A good fit is shown by a *p* > 0.05 (i.e., not significant). This test is not always reliable on large samples because it is very sensitive to size. **(ii)** The comparative fit index (CFI) estimates to what extent the tested model is better than the independence model (i.e., the model where each of the factors are independent and uncorrelated). Ideally, this score should be higher than 0.95 (perfect) or at least better than 0.90 (acceptable). **(iii)** The Tucker-Lewis index (TLI) is very close to CFI, it evaluates the degree to which the model improves the fit with respect to the independence model. For example, if the TLI is equal to 0.95, the studied model improves the fit by 95% compared to the independence model. As CFI, this score should be higher than 0.95 or at least better than 0.90. **(iv)** The root mean square error of approximation (RMSEA) is the index of poor fit of the tested model. The smaller the RMSEA, the better the goodness of fit. It is thus preferable to have the smallest possible value of RMSEA (preferably less than 0.05). **(v)** Finally, we can also look at the standardized root mean squared residual (SRMR), this latter must be < 0.08. It measures the difference between the correlation matrix of the observed sample and the matrix predicted by the model.

### 2.4. Quantification of the impact of the different factors on BCI acceptability

#### 2.4.1. Important factors in each category of our model: Mediation analysis

As one of our main aims was to determine the most influential determinants of PU, PEOU, and BI, we chose to perform mediation analyzes. This analysis is a rearranged linear regression, its objective being to decompose and quantify the total effect of a cause X on a response variable Y into a direct effect and an indirect effect through the mediator(s). This method was very relevant in our context: we had an acceptability model with independent variables, moderators (PU and PEOU) and a target variable (BI). We did one mediation analysis per category in our model (i.e., ***social influence, individual*** ***differences, facilitating condition, and system characteristics***—these categories are depicted in Section 3.1), in order to see which factors had the most impact in each of them. This analysis was also an interesting step to enable us to propose a shorter and simplified version of our model and questionnaire in the future, one with only the most relevant variables. The mediate library from R “psych” package was used (Revelle, 2021).

#### 2.4.2. Important factors independently of structure of the model: Random forest algorithm

After mediation analyzes, we wanted to do additional observations that do not depend on the architecture of our proposed model. We thus opted for the random forest (RF) algorithm. The principle of this algorithm is to randomly build multiple decision trees and train them on different subsets of our data. Thus, instead of trying to obtain an optimized method at once, we generate several predictors before pooling their different predictions. The final estimation is obtained, in the case of a regression as for this study, by taking the average of the predicted values. RF algorithms have the advantage of being non-parametric tests allowing the combination of quantitative and qualitative data, and making it possible to identify the factors associated with the bigger weights.

#### 2.4.3. Intensity of the connections between factors: Correlation analysis

After using the RF algorithm, we looked at the correlations between the most salient factors which stood out. Our objective was to see if the correlations between these factors were rather positive or negative in order to understand the meaning of their relationship (RF algorithm does not provide the strength of the connection between factors and target variable). To build the correlation matrix, a non-parametric method (Spearman's coefficient) was applied (Kowalski, 1972), and p-values were adjusted using the Bonferroni method.

## 3. Results

In this section, we present the acceptability model we built, the results of the questionnaire, our analyzes to validate these latter and finally those for quantifying the impact of the different factors on BCI acceptability.

We performed these analyzes using data from Qualtrics, after measuring the average score of each factor for every respondent (as explained in Section 2.2, a factor is measured by several questions which we had to average). For the qualitative/categorical variables, we calculated the number of occurrences of the sub-modalities.

### 3.1. Design of the acceptability model

**Figure 2:**
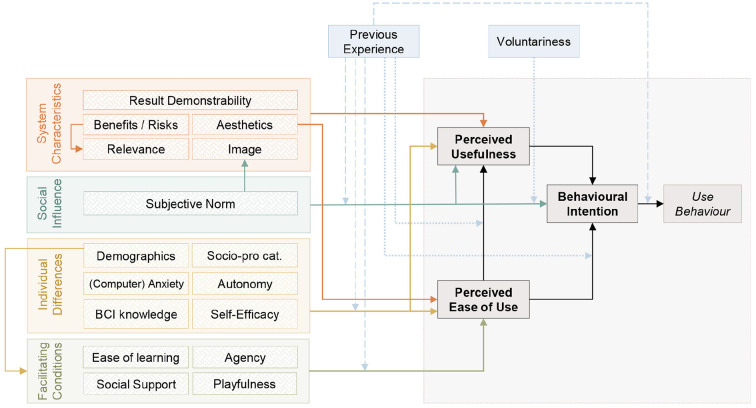
Representation of the tentative model of acceptability of BCIs for motor rehabilitation after stroke. On the right (in gray) are the target factors from TAM3 namely, PU, PEOU and BI. On the left are the four categories of factors that may influence the target factors: ***system characteristics*** (orange), ***social influence*** (turquoise), ***individual*** ***characteristics*** (yellow) and ***facilitating conditions*** (green). Each category includes one or more factors, themselves assessed in the questionnaire by 3–5 items. Solid arrows represent the potential influence of those categories on the target factors. Finally, on top, two moderators are represented in blue. Those factors moderate the effect of the different categories on the target factors. Dotted lines represent moderation effects presented in TAM3 while broken lines represent effects depicted in UTAUT2 (or in both).

We introduce here the theoretical model of acceptability dedicated to BCI-based post-stroke rehabilitation procedures that we have created. To design this model, we selected factors from those of the existing models presented in Table 1, using studies on BCIs to estimate their suitability in our context. To these current factors, we have added new ones that seem particularly relevant to BCIs, still basing ourselves on the BCIs literature. We present in this section the factors we included in our model (Table 2 contains definition and justifications of our choices) and its structure (Figure 2).

**Table 2:** Factors included in our acceptability model for BCIs in post-stroke motor rehabilitation.

**Factors**	**Validated models**	**Explanations**
**Target factors**		
Behavioral intention-BI	TAM3-UTAUT2-CUE	BI designates the prediction of an user's intention to use a technology. This is the key factor in acceptability models: a strong intention is the sign of a good acceptability of the system. BI is influenced by all other factors to greater or lesser degrees.
Perceived usefulness-PU	TAM3-UTAUT2-CUE	Definition: PU is equivalent to “performance expectancy” in UTAUT. It corresponds to the user's belief level of the fact that the use of the technology will improve them performance.
Perceived ease of use-PEOU	TAM3-UTAUT2-CUE	PEOU is equivalent to “effort expectancy” in UTAUT. It consists in the degree of belief to which using the system will require little or no effort (Terrade et al., 2009).
		**Justification:** In the literature, these two factors and the way they interact with each other are essential in the prediction of BI. Moreover, they are present in all of the papers that assessed the acceptability of BCIs using validated questionnaires of acceptability (Pillette et al., 2022).
**Characteristics of the system**		
Image	TAM3-UTAUT2	Definition: Image refers to the social image reflected when using a technology (social status, positive/negative perception by society). It is part of the process of social influence in TAM3 and UTAUT2, but we have chosen to introduce it within the characteristics of the system because BCIs can have different physical/material aspects, which probably influences the image.
		**Justification:** To our knowledge, the role of social norms on the intention to use BCIs has not really been studied. We nevertheless believe that there is an interest in verifying whether this factor plays or not a role on acceptability given the large number of categories of people who gravitate around post-stroke subjects—as we said for subjective norm.
Relevance	TAM3	Definition: This is the relevance from a scientific standpoint (i.e., relevance according to experts, to the latest science advances). We hypothesize that the scientific relevance of the BCI can be called into question when the benefits on risk ratio is low.
		**Justification:** Due to the large number of false beliefs about BCIs (Bocquelet et al., 2016), there is a strong interest in taking this factor into account in the context of rehabilitation with these technologies.
Result demonstrability	TAM3	Definition: This factor assesses the degree to which an individual believes that the results of using technology are tangible, observable and communicable (Moore and Benbasat, 1991).
		**Justification:** It is particularly interesting to evaluate it for BCIs in rehabilitation in order to understand how clear the information provided to the subject seems to them.
Visual aesthetics	CUE	Definition: The factor is introduced by CUE among the non-instrumental qualities of a system which “concern the look and feel of the system [...] non-instrumental qualities result from its appeal and attractiveness” (Thüring and Mahlke, 2007). Visual aesthetics refers more particularly the physical appearance of the system.
		**Justification:** Given that BCIs do not have a very attractive or aesthetic appearance, it can be assumed that this will have a possible impact on their acceptability, both in the user's relationship to the system—in particular their anxiety—and in the reflected social image.
Benefits/risk ratio	–	Definition: We decided to introduce this ratio as in medical context comparing a new therapy to conventional therapies is important; especially since learning how to use a BCI can be costly in time. In addition, the risk/benefit ratio is a common measure in the medical community (Edwards et al., 1996).
		**Justification:** Our choice is in accordance with the conclusion of Wolbring et al. (2013) who notes that “the clinical viability of BMI [brain-machine interface] technology for disabled people is determined by a cost (surgical risks, financial accessibility, reliability) benefit (improvement of quality of life) analysis.”
**Social influence**		
Subjective norm	TAM3-UTAUT2	Definition: “The degree to which an individual perceives that most people who are important to him think he should or should not use the system” (Fishbein and Ajzen, 1977; Venkatesh and Davis, 2000).
		Subjective norm is supposed to influence *image* (TAM3). This link would reflect the effect of the so-called identification process, i.e., when the subjects accept the use of a technology in order to maintain a positive relationship with the social group to which they belong (Kelman, 1958).
		**Justification:** Subjective norm is relevant for BCIs in clinical settings because patients are often assisted/supported by many people (close relations, nursing staff, other patients, etc.) who can influence their choices.
**Individual differences**		
Age and gender	UTAUT2	**Justification:** The user's personal characteristics were introduced as moderator variables in UTAUT (Venkatesh et al., 2003). We have chosen to keep them as classic factors (i.e., not just moderators), in order to see whether or not, in our context, they directly influence PU, PEOU or BI.
		In UTAUT2, age and gender influence also the ***Facilitating conditions*** category.
Computer anxiety	TAM3	Definition: “The degree of an individual's apprehension, or even fear, when she/he is faced with the possibility of using computers” (Venkatesh, 2000; Venkatesh and Bala, 2008).
		**Justification:** Computer anxiety (here anxiety regarding BCIs) is a relevant factor as it has been shown that fear of BCIs affects user performance (Burde and Blankertz, 2006; Nijboer et al., 2010; Witte et al., 2013). This apprehension toward the use of BCIs can be compared to a feeling of computer anxiety (Jeunet et al., 2016).
Computer self-efficacy	TAM3	Definition: “The degree to which an individual believes that he or she has the ability to perform a specific task/job using the computer” (Compeau and Higgins, 1995; Venkatesh and Bala, 2008).
		**Justification:** The study (Nijboer et al., 2008) shows that participant's high confidence in their training success leads to a better control over the BCI. Conversely, this same study found that a high level of fear of incompetence is associated with much lower control capacities.
General anxiety	–	Definition: Anxiety corresponds to waiting more or less consciously for future dangers or problems. In post-stroke subjects, the overall pooled estimate of anxiety disorders assessed by rating scale is 25% (Burton et al., 2013).
		**Justification:** This factor was chosen because the measure of general anxiety enables to differentiate anxiety generated by BCIs from anxiety disorder; the former can be softened by the context of use whereas the second is less controllable.
Autonomy	–	Definition: Autonomy is defined as people's concern “for their individuality, their independence, their efforts to achieve a goal, as well as a low concern for others” (Husky et al., 2004).
		**Justification:** A BCI study showed a strong correlation between self-reliance and mental imagery (MI) BCI performance (Jeunet et al., 2015). Self-reliance is an item of the 16PF5 questionnaire (Cattell and Cattell, 1995) which is an equivalent to autonomy as it measures the capacity to act in an autonomous way. We therefore believe that autonomy is a factor to include in order to better understand the attitude toward BCIs.
**Facilitating conditions**		
Agency	–	Definition: “The sense that I am the one who is causing or generating an action” (Gallagher, 2000).
		**Justification:** We introduce this factor in our model in light of BCIs studies. Indeed (Jeunet et al., 2016) showed that a low feeling of control and agency leads to poor performance with BCIs and Dussard et al. (2022) had preliminary results implying that a greater agency can improve BCI performances.
Computer playfulness	TAM3-UTAUT2-CUE	Definition: Introduced in TAM3, this factor is inspired by the concept of microcomputer playfulness presented by Martocchio et al., it “represents a type of intellectual or cognitive playfulness [...] an individual's tendency to interact spontaneously, inventively, and imaginatively with microcomputers” (Martocchio and Webster, 1992).
		**Justification:** We think that in BCI context, a pleasant and playful interface can reduce the apprehension and the fear felt toward the system. The consideration of playfulness is common in motor rehabilitation (Korn and Tietz, 2017), but also in the field of BCIs, in particular in a logic of gamification and of combination of virtual reality and BCI (Ron-Angevin and D́ıaz-Estrella, 2009; Wang et al., 2022).
Ease of learning	–	Definition: This factor is inspired from the System Usability Scale (SUS) (Brooke, 1986)—the questionnaire measures ease of use, but some questions are in link with the learning. We define it as the degree to which a person believes that learning how to use a system will be effortless.
		**Justification:** This is particularly interesting for BCIs as learning to use them is not easy (Benaroch et al., 2021), regardless of the level of expertise in the use of others technologies. For example, Pasqualotto et al. (2011) found that computer skills did not influence BCI—type P300 Speller—performance.
Social support	–	Definition: Social support is a new item which we believe may help to adapt BCIs for rehabilitation protocols. It is the degree to which an individual feels they need a human presence for BCI use and the context in which they would need it.
		**Justification:** The study of Pillette et al. (2020) shows that the presence of a tangible companion, who provides social and emotional support, has positive effects for certain participant profiles. For rehabilitation, we find it especially relevant to measure social support since research on BCIs in clinical situation is conducted both in hospital and at home (Leeb et al., 2013; Zulauf-Czaja et al., 2021).
**Moderators**		
Previous experience	TAM3-UTAUT2	Definition: This moderator concerns both taking into account the user's experience with BCIs and with new technologies in general. It moderates the effects that ***social influence**, **individual differences*** and ***facilitating conditions*** have on the target factors as well as the influence that PEOU has on PU and BI.
		The effect of PEOU on BI is lessened (TAM3 and UTAUT2) while the one of PU is strengthened (TAM3) with experience. In UTAUT2, it is also suggested that this *previous experience* factor moderates the effect of BI on the final use of the technology: the more experienced the user, the less influence BI has on actual use of the technology.
		**Justification:** It is not new to take experience into consideration in BCIs studies, it is for example the case in Pasqualotto et al. (2011) and Randolph (2012).
Voluntary	TAM3-UTAUT2	Definition: This factor expresses if the use is mandatory or voluntary, this can potentially affect acceptability factors. It has been suggested to influence directly BI (TAM3).
		In our questionnaire, we could not really measure this factor since it was not followed by an actual use of a BCI. We have therefore adapted the wording of the questions so that they correspond to a supposedly voluntary use (e.g., “If I had the possibility…”).
		**Justification:** Some researchers have found, for example, that the role of ***social influence*** is significant when use is constrained, and not when it is voluntary (Wills et al., 2008). These results are not always found (Wang et al., 2009; Schaupp et al., 2010), so it is relevant to observe the role of this moderator for BCIs.

Each factor is classified into a category: ***social influence***, ***individual differences***, ***facilitating conditions***, and ***system characteristics***. These categories are inspired by TAM3 (Venkatesh and Bala, 2008) and UTAUT2 (Venkatesh et al., 2012). ***Social*** ***influence***, as defined in the TAM2 and 3, is the influence of an individual's relatives and social group on their choice of whether or not to adopt a system. It is a determinant of PU and BI. Its effect on BI and PU decreases with experience (according to TAM3 and UTAUT2, and only to TAM3, respectively). ***Individual differences*** is a category which groups the user personal characteristics (socio-demographic information, cognitive traits and personality). Its factors are determinants of PU and PEOU. We hypothesize that the weight of the factors of this category decrease with experience as the effect of computer anxiety on PU and PEOU decreases with experience (TAM3). ***Facilitating*** ***conditions*** brings together the factors related to the material, organizational and/or human conditions that facilitate the use of a technology (Février, 2011). This category is a determinant of PEOU (TAM3). Its impact is lessened while users acquire experience with the technology as their dependence toward external support will be reduced (Alba and Hutchinson, 1987). Finally, ***system characteristics*** is a category related to the instrumental cognitive process introduced by the TAM2. It is the mental representation developed by the user to judge what the use of a technology can bring them in relation to their objective(s) (relevance of the system, perceived quality, etc.) (Terrade et al., 2009). This category influences PU (TAM3) and in addition, among this category, *visual aesthetics* also influences PEOU because this factor comes from the CUE model which assumes that its effect is not limited to PU.

### 3.2. Results of the questionnaire

#### 3.2.1. Participants

We managed to obtain a set of *N* = 753 respondents to our questionnaire based on the model. This sample was representative of the composition of the adult population in France. We provide the socio-demographic details in Table 3.

**Table 3:** Socio-demographic information of the respondents of the questionnaire.

	**Objective %**	**Objective N**	**Obtained %**	**Obtained N**
**Source: National Institute of Statistics and Economic Studies (INSEE)-2016 to 2019**				
**Gender**				
Male/Female	47.4%/52.6%	315/350	48.7%/51.3%	367/368
**Age (years)**				
20–24	7.7%	51	10.2%	77
25–34	16.2%	108	17.4%	131
35–44	16.9%	112	18.2%	137
45–54	17.7%	118	16.3%	123
55–64	16.4%	109	14.9%	112
65 and more	25.1%	167	23.0%	173
**Socio-professional categories**				
Own account workers (agriculture, craftsperson, shopkeeper, company head)	4.6%	31	5.2%	39
Higher managerial, administrative and professional occupation	10.9%	72	11.2%	84
Intermediate managerial, administrative, professional occupations	15.2%	101	15.1%	114
Lower supervisory and technical occupations	29.4%	196	27.2%	205
Retired	28.9%	192	28.6%	215
Others unemployed	11.0%	73	12.7%	96
**Region**				
Ile-de-France	20.8%	138	19.3%	145
North-west	21.7%	144	23.5%	177
North-east	21.0%	140	22.2%	167
South-west	10.7%	71	10.2%	77
South-east	25.8%	172	24.8%	187
Total	100.0%	665	100.0%	753

The gray is for objective values whereas the violet is for obtained values.

95.8% of our sample had never used BCIs—including 68.7% that didn't hear about BCIs before this questionnaire. This lack of knowledge was consistent with our objectives because it is more relevant to have novice users when measuring acceptability—as it should be before any interaction with the technology. In consequence, we didn't discuss in detail the *previous experience* moderator of our model in this paper, as we didn't have enough expert respondents to differentiate inexperienced/experimented users.

#### 3.2.2. Descriptive analysis

In Table 4, we present the mean scores of each quantitative factor, and the percentages for categorical factors. None of the factors was associated with a score below 5/10, which reflects globally positive feelings and well-perceived BCIs among the respondents. Indeed, regarding the target factors, BI2 had a mean of 8.21 (*SD* = 1.67), for PU2 it was 8.28 (*SD* = 1.57) and for PEOU the mean was 7.17 (*SD* = 1.57).

**Table 4:** Results from general public questionnaire.

**Factors**	**Type**	**Mean**	***SD***
**System characteristics**			
Result demonstrability	Quantitative	6.84	1.68
Benefits/risks ratio	Quantitative	7.27	1.51
Relevance	Quantitative	8.03	1.48
Image	Quantitative	6.10	2.17
Visual aesthetic	Quantitative	6.62	1.89
**Social influence**			
Subjective norm	Quantitative	7.38	1.71
**Individual differences**			
Autonomy	Quantitative	7.40	1.46
General anxiety	Quantitative	5.49	1.87
Computer anxiety	Quantitative	6.34	2.50
		**Condition**	**%**
Computer self-efficacy	Qualitative	Alone. independently	23.11%
*Usage conditions if BCI installed and explained*		Alone. if has experience with a similar technology	11.95%
		Alone. with support from a virtual companion	36.65%
		Only with human guidance and presence	28.29%
Social support	Qualitative	Independently. alone at home	32.80%
		In the presence of a health professional	47.70%
		Alone. but in a health facility	19.50%
BCI Knowledge	Qualitative	Yes-has already used a BCI	3.98%
		Yes-but has never used a BCI	27.09%
		No	68.66%
		**Mean**	**SD**
**Facilitating conditions**			
Agency	Quantitative	6.29	1.65
Playfulness	Quantitative	6.90	1.79
Ease of learning	Quantitative	5.96	1.62
**Characteristics of the system**			
Result demonstrability	Quantitative	6.84	1.68
Benefits/risks ratio	Quantitative	7.27	1.51
Relevance	Quantitative	8.03	1.48
Image	Quantitative	6.10	2.17
Visual aesthetic	Quantitative	6.62	1.89
**Main target factors**			
BI	Quantitative	7.87	1.72
PU	Quantitative	7.87	1.63
PEOU	Quantitative	7.17	1.57
PU 2	Quantitative	8.28	1.57
BI 2	Quantitative	8.21	1.67

Scores for the quantitative variables were continuous from 0 to 10 (*strongly disagree* to *strongly agree*).

As explained in Section 2.2, our questionnaire contained two videos. We wanted to verify if, depending on the richness of information provided to people about BCIs in rehabilitation (possibilities of use, expected results, etc.), the factors that most impact BI and PU are or not the same. In this aim, we compared the means of the two paired samples (before/after the video explaining how BCIs could be integrated in stroke rehabilitation, i.e., video 2) for BI and PU. Wilcoxon test with Bonferroni correction was used, it evaluated if there was a significant difference between the values of PU1/PU2 and BI1/BI2. We didn't measure PEOU twice, even if it is one of the main determinant of BI in literature, because the users' viewpoint about the functioning of BCI remains the same as long as they had never have the opportunity to actually test the interface before.

**Figure 3:**
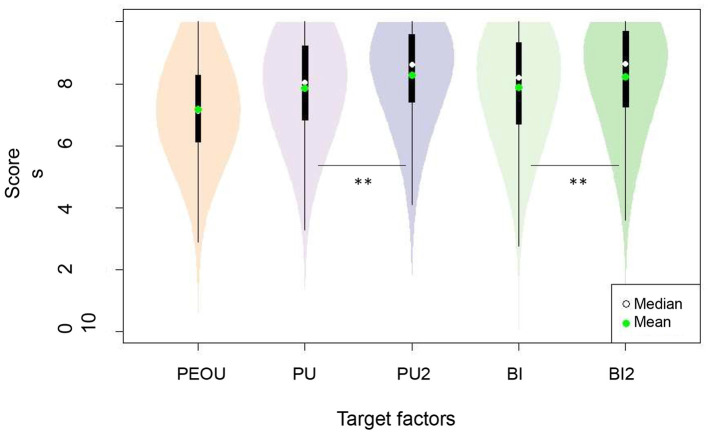
Distribution of the scores as a function of the different target factors. Paired Wilcoxon test: for the [PU1-PU2] and [BI1-BI2] pairs, we obtained a *p* < 0.001 (with Bonferroni correction), each factor was therefore significantly different. The ** symbol indicated to show that the factors were significantly different (i.e., PU/PU2 and BI/BI2).

Wilcoxon test showed that the scores of BI1 and PU1 were significantly different from BI2 and PU2 respectively (see Figure 3). As pointed out in Table 4, the means were higher after the video, which seemed to have a positive impact on the respondents' standpoint about BCI (*Before video 2:* PU1 mean = 7.87, *SD* = 1.63/BI1 mean = 7.88, *SD* = 1.73. *After video 2:* PU2 mean = 8.28, *SD* = 1.57/BI2 mean = 8.21, *SD* = 1.67). The score of PEOU was also high (mean = 7.17, *SD* = 1.57).

### 3.3. Validation of the structures of the model and questionnaire

#### 3.3.1. Coherence of the factors: Cronbach's alpha

Cronbach's alpha analyzes (Table 5) show that 13/17 factors had a satisfactory internal consistency, with scores comprised between 0.72 and 0.97. Among the four other factors, the scores were the following: 0.5 (*agency*), 0.52 (*autonomy*), 0.57 (*ease of learning*) and 0.62 (*benefits/risk ratio*).

**Table 5:** Cronbach's alpha reliability values for the questionnaire based on our acceptability model.

**Categories**	**Factors**	**Alpha coefficient**
System characteristics	Benefits/Risk ratio	0.62
	Result demonstrability	0.72
	Image	0.80
	Visual aesthetics	0.83
	Relevance	0.91
Social influence	Subjective norm	0.83
Individual differences	Agency	0.50
	Autonomy	0.52
	General anxiety	0.77
	Computer anxiety	0.91
Facilitating conditions	Ease of learning	0.57
	Playfulness	0.83
Target factors	PEOU	0.83
	PU 1	0.91
	BI 1	0.95
	PU 2	0.95
	BI 2	0.97

The closer the value is to 1, the better the internal consistency of the factor. It is estimated that below 0.7, consistency is weak. Orange is for factor below 0.7 (i.e., coefficient not very satisfactory), green is for factors between 0.7 and 0.95 (i.e., good coefficient), dark green is for factor above 0.95 (i.e., coefficient very high, which may be a sign of redundancy in the items of the factor).

#### 3.3.2. Structure of the model: Confirmatory factor analysis

Regarding the CFA results, we obtained a *p*-value of 0.0 for the chi-square test. This means that the hypothesis of the perfect fit of the model to our data is rejected. Nevertheless, this can be explained by the large size of our sample. The comparative fit index (CFI) value was 0.913 and the Tucker-Lewis index (TLI) value 0.897, which indicates a good fit between the model and the data. Indeed, these scores mean that our model is better than the independence model. The RMSEA, which is the index of poor adjustment of the model, should ideally be below than 0.05. Results indicated a value of 0.059, with a confidence interval ranging from 0.056 to 0.062. It was thus close to the expected value. Finally, our SRMR was 0.076 (i.e., < 0.08, as expected as this test assesses the divergence between observed and expected correlations).

### 3.4. Quantification of the impact of the different factors on BCI acceptability

#### 3.4.1. Important factors in each category of our model: Mediation analysis

Table 6 presents the different results we obtained following the mediation analyzes. It should be noted that the categorical factors are not presented here (demographics, self-efficacy, BCI knowledge and social support), we studied only the quantitative variables because our categorical variables were not binary, so it was not adapted to this method. Nevertheless, they are not left out, we present an analysis included them with RF algorithm in Section 3.4.2.

**Table 6:** Table of scores for mediation analysis (only with quantitative factors).

**Category**	**Independent variable (IV)**	**Total effect**	**Effect IV-MV**		**Effect MV-DV**		**Direct effect**	**Indirect effect**	**BC 95% CI of indirect effect (bootstrap : nb iterations = 500)**
			**PU2**	**PEOU**	**PU2**	**PEOU**			
System Characteristics - PEOU	Visual aesthetic	0.32^**^	–	0.38^**^	–		0.04	0.28	[0.22; 0.34]
		*p*= 3.19e-22		*p* = 2.48e-43		0.72^**^	*p* = 1.47e-01		
	Image	0.09	–	0.17^**^		*p* = 3.29e-76	−0.03	0.12	[0.22; 0.5]
		*p* = 8.97e-04		*p* = 8.81e-14			*p* = 1.26e-01		
System characteristics-PU2	Relevance	0.54^**^	0.65^**^	–		–	0.04	0.50	[0.41; 0.60]
		*p* = 6.24e-51	*p* = 6.24e-83				*p* = 1.78e-01		
	PEOU	0.19^**^	0.2^**^	–			0.04	0.15	[0.09; 0.21]
		*p* = 2.67e-11	*p* = 2.77e-14				*p* = 5.17e-02		
	Benefits/Risks ratio	0.23^**^	0.15^**^	–	0.77^**^		0.11^**^	0.12	[0.06; 0.19]
		*p* = 1.73e-13	*p* = 1.51e-08		*p* = 1.08e-102		*p* = 4.12e-06		
	Result demonstrability	0.14^**^	0.04	–			0.11^**^	0.03	[0.0; 0.07]
		*p* = 1.73e-08	*p* = 5.15e-02				*p* = 4.18e-09		
	Visual aesthetic	0	−0.02	–			0.02	−0.01	[-0.04; 0.01]
		*p* = 8.34e-01	*p* = 3.06e-02				*p* = 2.17e-01		
	Image	−0.02	−0.01	–			−0.01	−0.01	[-0.03; 0.01]
		*p* = 2.76e-01	*p* = 5.26e-01				*p* = 3.70e-01		
Social influence	Subjective norm	0.63^**^	0.58^**^	0.57^**^	0.89^**^	0.06	0.08^**^	0.55	[0.47; 0.61]
		*p* = 2.48e-91	*p* = 5.68e-86	*p* = 1.88e-80	*p* = 4.95e-198	*p* = 6.04e-03	*p* = 2.16e-06		
Individual differences	Autonomy	0.34^**^	0.36^**^	0.33^**^			−0.01	0.35	[0.27; 0.42]
		*p* = 1.32e-19	*p* = 1.24e-23	*p* = 8.39e-19			*p* = 4.85e-01		
	Computer anxiety	0.27^**^	0.22^**^	0.15^**^	0.89^**^	0.09^**^	0.06^**^	0.21	[0.16; 0.26]
		*p* = 4.42e-33	*p* = 4.94e-26	*p* = 1.68e-12	*p* = 1.17e-202	*p* = 5.87e-06	*p* = 2.27e-10		
	General anxiety	−0.05	−0.04	−0.04			−0.01	−0.04	[−0.09; 0.02]
		*p* = 1.20e-01	*p* = 1.43e-01	*p* = 1.34e-01			*p* = 6.74e-01		
Facilitating conditions	Playfulness	0.41^**^	0.36^**^	0.39^**^		0.01 *p* = 5.50e-01	0.09^**^	0.32	[0.25; 0.39]
		*p* = 1.69e-25	*p* = 3.25e-21	*p* = 1.38e-30			*p* = 3.09e-06		
	Agency	0.28^**^	0.25^**^	0.06	0.88^**^		0.05	0.22	[0.16; 0.29]
		*p* = 3.76e-13	*p* = 8.75e-12	*p* = 5.87e-02	*p* = 4.12e-203		*p* = 2.88e-03		
	Ease of learning	0	−0.01	0.29			0.01	−0.01	[−0.08; 0.06]
		*p* = 9.64e-01			*p* = 6.90e-01	*p* = 6.19e-19			*p* = 5.78e-01
Main target factors	PEOU	0.73^**^	0.69^**^	–	0.92^**^	–	0.09^**^	0.64	[0.58; 0.71]
		*p* = 9.94e-105	*p* = 4.25e-110		*p* = 1.02e-220		*p* = 1.03e-05				

The mediators variable were PU2 and PEOU, the dependant variable was BI2. ^**^p < 0.001–BC, bias corrected; DV, dependent variable; MV, mediator variable; PU, perceived usefulness; PEOU, perceived ease of use. Color differences are for ease of reading the table, no special meaning.

**Figure 4:**
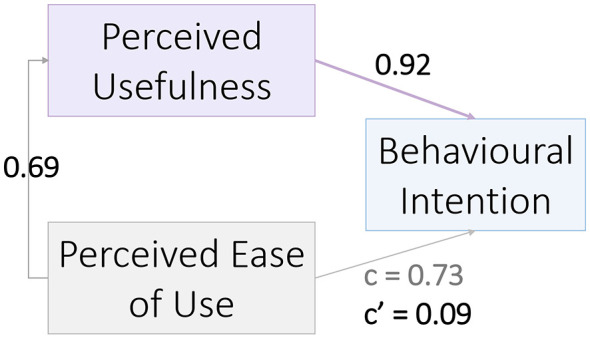
Mediation analysis for the target factors: **Behavioral intention** (BI2), **Perceived usefulness** (PU2) and **Perceived ease of use** (PEOU). *R*^2^ = 0.86 (*p* < 0.001). c, total effect of PEOU on BI2; c', direct effect of PEOU on BI2; c-c', indirect effect of PEOU on BI2 through PU2.

Figure 4 shows that the BI was mainly influenced by PU2 (effect: 0.92, *p* < 0.001), the weight of the PEOU being much lower (direct effect: 0.08, standard error *SE* = 0.02, *p* < 0.001; indirect effect: 0.65, *SE* = 0.03, CI = [0.58, 0.71]), i.e., PEOU had a low effect on BI2 but a significant effect on PU2.

Concerning the other categories of our model, our results revealed that for the ***individual*** ***differences***, *autonomy* was the most influential factor on BI, but this effect was moderate (*c* = 0.34, *p* < 0.001), it equally impacted PU2 and PEOU (respectively, 0.36 and 0.33, with *p* < 0.001) (quality of the model: *R*^2^ = 0.87, *p* = 0.0).

For *social influence, subjective norm* had a similar and moderate impact on both PU2 and PEOU (respectively, 0.58 and 0.57, with *p* < 0.001). The influence on BI2 was rather high (c = 0.63, *p* < 0.001) (quality of the model: *R*^2^ = 0.87, *p* < 0.001).

For *characteristics of the system*, we did two analyses: (i) one with only PEOU as mediator, and factors present before video 2 (PU1 was not included since we chose to focus on PU2); (ii) the second only with PU2 as mediator, and factors present before and after video 2 (PEOU was among these factors since, as shown in Figure 2, it influences PU). **(i)** shows that *visual aesthetics* was the most—but weak—influential factor on PEOU (0.38, with p < 0.001). The total effect of *visual aesthetics* on BI2 was low: c = 0.32 (*p* < 0.001) (quality of the model: *R*^2^ = 0.48, *p* < 0.001). On the other hand, (ii) revealed that *relevance* was the most influential factor on PU2 (0.65, with *p* < 0.001). Its total effect on BI2 was c = 0.54 (*p* < 0.001) (quality of the model: *R*^2^ = 0.88, *p* < 0.001).

Finally, for *facilitating conditions*, the variable with most impact was *computer playfulness*, it equally impacted PU2 and PEOU (respectively, 0.36 and 0.39, with *p* < 0.001). The influence of *computer playfulness* on BI2 was moderate (c = 0.41, *p* < 0.001) (quality of the model: *R*^2^ = 0.87, *p* < 0.001). Additional figures of mediation analysis are available in **Supplementary material 2**.

#### 3.4.2. Important factors independently from the structure of the model: Random forest algorithm

We ran the RF algorithm in order to explain the values of our 3 target factors: BI, PU and PEOU. RF algorithms have the advantage of enabling analyzes with qualitative and quantitative variables at the same time so we used it on all our factors. Table 7 presents the important variables of BI2, PU2, and PEOU. The ordering of these variables enabled us to determine which of our factors explained the best the scores of these target factors. The three most important variables for each of them were:

- **For BI2:** *PU2* followed by *relevance* and *benefits/risk ratio*. PU2 was in large predominance (value = 100) in comparison to the other (37.4 and 30.3, respectively). The quality of the prediction was high: 86.09% of the variance is explained.
- **For PU2:** *Relevance, PEOU* and *benefits/risk ratio*. *Relevance* was much more influential than the others (value = 100, vs. 33.5 and 33.4, respectively). The quality of the prediction was still quite high with 79.64% of the variance is explained.
- **For PEOU:** *Ease of learning, computer playfulness* and *subjective norm*. The values were less disparate: 100, 83.2, 80.9, respectively. But the prediction had a lower quality with 57.76% of the variance is explained.

**Table 7:** The 20 most influential factors for each target factor (BI, PU, and PEOU) based on the RF algorithm.

**Perceived ease of use (PEOU)**		**Perceived usefulness (PU) 2**		**Behavioral intention (BI) 2**	
**Ease of learning**	**100.00**	**Relevance**	**100.00**	**PU2**	**100.00**
**Playfulness**	**83.21**	**PEOU**	**33.54**	**Relevance**	**37.44**
**Subjective norm**	**80.86**	**Benefits/risk ratio**	**33.42**	**Benefits/risk ratio**	**30.25**
Visual aesthetic	66.46	Subjective norm	31.96	Subjective norm	29.56
Image	41.11	Result demonstrability	19.11	Result demonstrability	28.02
Agency	32.73	Playfulness	17.11	Playfulness	27.67
*Age*	*24.75*	Computer anxiety	14.43	PEOU	24.94
Computer anxiety	22.98	Autonomy	12.59	Computer anxiety	22.83
General anxiety	19.28	Visual aesthetic	12.34	Autonomy	17.37
Autonomy	17.19	Ease of learning	12.02	Agency	17.29
Experience-pleasure	12.27	Experience-confidence	10.60	Visual aesthetic	16.86
*Gender-Women*	*11.78*	Experience-pleasure	10.51	Ease of learning	16.05
*Self-efficacy-3*	*11.77*	Agency	10.03	Image	15.17
*Self-efficacy-2*	*8.80*	Image	8.01	Experience-pleasure	12.88
*Employees*	*8.49*	*Gender-Women*	*6.27*	Experience-confidence	12.16
*Intermediate occupations*	*7.58*	General anxiety	5.78	*Self-efficacy-2*	*10.60*
Experience-confidence	7.28	*Lower occupations*	*5.20*	*Self-efficacy-1*	*10.30*
*Higher occupation*	*6.89*	*Higher occupation*	*5.19*	*Without activity*	*9.68*
*Without activity*	*6.24*	*Intermediate occupations*	*4.73*	*Employees*	*8.70*
*Social support-1*	*6.11*	*Self-efficacy-3*	*4.56*	*Intermediate occupations*	*8.41*

The factors *Experience-pleasure* and *Experience-confidence* were about the pleasure and confidence of respondents toward the use of technologies in general. % Variance explained: BI2 = 86.09; PU2 = 79.64; PEOU = 57.76 [RF with 500 trees and cross-validation (5-fold)]. The importance values correspond to the mean decrease accuracy (%IncMSE). We scaled the values from 0 to 100 and ranked them in decreasing order to facilitate comparisons. Therefore, the most influential ones appear on top of the table. The bold values indicate the three most important variable for each target factor. The italic values indicate the categorical variables.

Categorical factors appeared to have only moderate, if not low impact on BCI acceptability. The age was the only one in the top 10 of most influential factors, for PEOU only.

**Figure 5:**
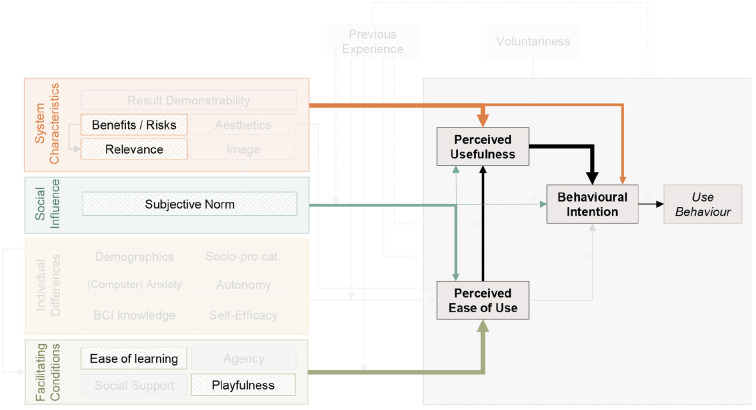
Representation of the factors of the tentative model of acceptability that influence the most the target factors. Boldest arrows link factors to the target factor they influence the most: *ease of learning* is the most influential factor for PEOU, *relevance* is the factor that has the highest impact on PU, itself being the most influential factor for BI. In addition, *relevance* also has a strong influence on BI. *Benefits on risk ratio* strongly influence both PU and BI. *Subjective norm* has a strong impact on PEOU (which was not expected based on the TAM3 and UTAUT2) and a medium impact on PU and BI. Finally, *computer playfulness* has a strong impact on PEOU.

To provide a visual overview of our analyzes results, we propose a simplified version of our initial model in Figure 5, keeping only the most significant factors.

#### 3.4.3. Intensity of the connections between factors: Correlation analysis

We ran correlation analyzes between all our quantitative factors (with Bonferroni correction). For seeks of readability, we show in Table 8 only the factors that had been identified as the most influential ones based on RF analyzes. Results reveal that all the correlation coefficients were positive. The strongest correlations were between BI2 and PU2 (0.92-Bonferroni-corrected *p* < 0.001), PU2 and relevance (0.89-Bonferroni-corrected *p* < 0.001), BI2 and relevance (0.86-Bonferroni-corrected *p* < 0.001), but all the factors were significantly and strongly correlated with the target factors.

**Table 8:** Spearman correlation analyzes for the most significant factors.

	**Subjective norm**	**Visual aesthetics**	**Playfulness**	**Ease of learning**	**PEOU**	**Result demonstrability**	**Benefits/risk ratio**	**Relevance**	**PU2**
PEOU	0.59	0.54	0.63	0.6					
	*p* < 0.001	*p* < 0.001	*p* < 0.001	*p* < 0.001					
PU2	0.63	0.39	0.55	0.37	0.66	0.69	0.74	0.89	
	*p* < 0.001	*p* < 0.001	*p* < 0.001	*p* < 0.001	*p* < 0.001	*p* < 0.001	*p* < 0.001	*p* < 0.001	
BI2	0.65	0.41	0.59	0.40	0.65	0.73	0.75	0.86	0.92
	*p* < 0.001	*p* < 0.001	*p* < 0.001	*p* < 0.001	*p* < 0.001	*p* < 0.001	*p* < 0.001	*p* < 0.001	*p* < 0.001

*p*-values adjusted with Bonferroni correction. The color shade is intended to show the value of the coefficient. The closer a coefficient is to 1, the more the color is intense.

## 4. Discussion

This paper provides the following contributions. First, we designed a first-of-its-kind model of acceptability of BCIs for motor rehabilitation after stroke. This model is based on the literature, and notably on three validated models: TAM3 (Venkatesh and Bala, 2008), UTAUT2 (Venkatesh et al., 2012), and CUE (Thüring and Mahlke, 2007). Second, we created, based on this model, a questionnaire to assess acceptability. This questionnaire follows the structure of the model and includes 3 to 5 items to measure each of the factors. The quantitative items are represented as analog visual scales for which participants move a cursor from “do not agree at all” to “perfectly agree.” The position of the cursor is then translated into a score (from 0 to 10). The scores of the items measuring the same factor are averaged in order to obtain a robust estimation of this factor that is not (or at least as little as possible) dependent on the (mis)understanding of the item or on the state of the person when they answered the question. We distributed this questionnaire to a sample representative of the adult population in France (*N* = 753). This large and representative sample theoretically ensures the reliability of our results. Third, we performed analyzes on the data obtained to validate the structure of the model. More specifically, we assessed on the one hand the internal consistency of the factors using Cronbach's alpha analyzes. This enabled us to verify the relevance and complementarity of the items used to assess each factor.

On the other hand, we performed a confirmatory factor analysis to evaluate the internal consistency of the questionnaire, or in other words the relevance of the structure of the model. Finally, this is the fourth contribution, we quantified the impact that the different factors had on our target factors (PEOU, PU and BI) in order to identify the factors that influence the most BCI acceptability in the general public. To do so, we used two complementary methods: mediation analyzes and regressions based on random forest algorithms. The first one assessed this influence by taking into account the structure of the model while the second was independent from that structure. Our results show that BCIs are associated with high levels of acceptability for motor rehabilitation after stroke in the general public, and that the intention to use these technologies in that context is mainly driven by the *perceived usefulness* of the system, itself being mostly influenced by some ***characteristics of the system***, and notably the *benefits on risk ratio* and *scientific relevance*. ***Facilitating conditions***, and notably *ease of learning* and *playfulness* are the main determinants of the *perceived ease of use*. Finally, the *subjective norm* significantly influences the three target factors. With this methodology and results, our study is a first step toward an in-depth consideration of acceptability of BCIs for motor rehabilitation procedures after stroke.

For now, the model and questionnaire, while (we hope) insightful, are not really usable in practice due to their length and complexity. We voluntarily used an exploratory approach by including all the potential influential factors in our model, considering that the literature in the field did not enable us to have strong a priori. The extensive dataset collected enabled us to obtain first indications of the most influential, and therefore most relevant-to-assess factors. More data should now be collected to (un)validate those first results and refine the estimation of the impact that each factor has on BCI acceptability. Our objective is, ultimately, to design a shorter and more usable questionnaire that will enable the prediction of BCI acceptability based on a few factors (and so few items). This prediction could provide scientists/clinicians some indications on how to adapt the procedure, including the instructions, tasks, feedback and training environment, to favor BCI acceptability. As mentioned in the introduction, high acceptability levels could serve as levers to improve BCI efficiency. A main result of this study is that, globally, acceptability levels in terms of behavioral intention seem to be very high in the general public (with an average score of 8.21/10). This is consistent with other BCI acceptability studies (Al-Taleb et al., 2019; Voinea et al., 2019; Benaroch et al., 2021) who reported average scores of 8.0/10 (Al-Taleb et al., 2019) and 6.0/7 (Voinea et al., 2019) for perceived usefulness.

The analysis of Cronbach's alpha revealed that all the factors from the TAM3, UTAUT2 and CUE questionnaire were associated with high-quality internal consistency, i.e., scores were between 0.70 and 0.95 (Cortina, 1993; Tavakol and Dennick, 2011). It was not the case for some of the factors we added (in complement of those from TAM3, UTAUT2 and CUE) to fit with specificities of BCIs, namely, *agency* (0.50), *autonomy* (0.52), *ease of learning* (0.57) and *benefits/risk ratio* (0.62). This might be due to inadequate wording of the items. It should be noted though that the items used to assess *autonomy* were directly extracted, word-by-word, from the “Sociotropy-autonomy scale” (SAS, Husky et al., 2004), while those used to measure *agency* and *ease of learning* are reformulations (adapted to the context of BCIs) of items from the French adaptations of the Sense of Agency Scale (F-SoAS, Hurault et al., 2020) and of the System Usability Scale (SUS, Gronier and Baudet, 2021), respectively. In the future, it would be relevant to i) collect more data to assess the significance of this result, and ii) lead investigations regarding the comprehension of those items by potential BCI users, and maybe to reword them to increase the internal consistency to the associated factors.

Regarding the extreme score of internal consistency obtained for BI2 (0.97), we hypothesize that it might be due to the repetition of the items. Indeed, when participants saw the same items a second time, it might have happened that they automatically put some scores without really thinking about it, due to perceived redundancy. This hypothesis is supported by the fact that PU2 was also associated with very high, internal consistency scores (0.95) -while still in the “acceptable range.” This high score might also be due to a ceiling effect on those dimensions. Indeed, PU1 and BI1 were already rated with high scores (7.87+/1.63 and 7.88+/–1.73, respectively). After the second video, participants globally increased their rating and gave PU2 scores of 8.28+/1.57, and BI2 scores of 8.23+/–1.69. Thus, the range of values attributed to the items of PU2 and BI2 was narrow, resulting in low variability and thereby very high consistency within those dimensions.

To conclude on the validity of the questionnaire, while it is certainly not perfect yet -we hope that the community will help us improving it by collecting data and suggesting modifications- analyzes have globally revealed i) good internal consistency (as measured by Cronbach's alpha scores) for a large majority of the factors, and ii) a relevant structure of the model (as measured by the confirmatory factorial analysis).

If we have a closer look at the factors influencing the intention to use BCIs, thanks to the random forest-based regression analyzes, we notice that our different analyzes are consistent, notably in showing no significant impact of ***individual*** ***differences***, including demographics (age, gender, socio-professional category) or cognitive/psychological profile (*autonomy, anxiety*, and *self-efficacy*). Yet, BCI studies have suggested an influence of those variables on BCI performance and learning (Burde and Blankertz, 2006; Nijboer et al., 2008, 2010; Witte et al., 2013; Jeunet et al., 2015). It might be possible that the weight of psychological variables such as *anxiety* or *autonomy* are stronger in persons with clinical conditions. It might also be the case that this influence is directly on efficiency as high levels of *anxiety* and low levels of *autonomy* and *self-efficacy* are detrimental for learning but does not alter acceptability. This reinforces the relevance of our approach consisting in optimizing acceptability in order to put the users/patients in the best conditions to favor learning despite their clinical condition, and thereby use acceptability as a lever to favor efficiency.

Behavioral intention is mainly influenced by the *perceived usefulness* of BCIs, itself being mainly determined by the perceived *scientific relevance* of the technology. This result highlights the importance of informing the population about BCIs, the way they function, and the level of scientific evidence regarding their clinical efficacy. This idea is strengthened by the significant increase of BI and PU scores following the second video in which the benefits of BCIs for motor rehabilitation after stroke are presented. In the same category of “***system characteristics***,” the *benefits on risk ratio* of the technology also seems to have a strong impact on acceptability. We hypothesize that this balance, as perceived by the user/patient, may have a moderator effect on the emphasis on *scientific relevance* that is objectively depicted by scientists and clinicians. Indeed, (irrational) fears or over-expectations may bias the balance making inaudible the scientific discourses.

Another main finding of this study is the influence that *subjective norm* has on the three target factors. This was expected for PU and BI. Nonetheless, if we refer to TAM3 and UTAUT2, this factor is not supposed to influence PEOU. In our results yet, this is on the latter that the *subjective norm* has the strongest impact. We hypothesize that the opinions of the patients' close ones, their technophilia and trust in science will, in the case of BCIs, not only play a role on the *perceived usefulness*, but will also contribute to emphasize or reduce apprehension toward the technology. This in turn may alter the *perceived ease of use* of the technology. In any case, the fact that ***social influence*** contributes in determining acceptability levels by acting on the three target factors reinforces the relevance of informing the general public, in which patients' relatives are included, to favor the acceptability and adoption of BCIs.

Finally, ***facilitating conditions***, and especially *ease of learning* and *playfulness*, are the main determinants of PEOU that, while not influencing BI directly, significantly impacts PU. We believe that this result should encourage us to keep in mind that instructions should be clear and training motivating when we design BCI procedures. This will enable patients to feel confident in their ability to use a BCI. Providing an engaging environment can also be a way to make training more accessible. These results are consistent with the guidelines for successful MI-BCI training (Roc et al., 2021). The question of the transferability of this result to populations of patients could be raised. Indeed, the general population, while they do not need to use BCIs for rehabilitation, may perceive BCIs as a “toy,” which could explain this result. In fact, *playfulness* has also been shown to increase the compliance of patients in the rehabilitation process in other fields (Burke et al., 2009; Korn and Tietz, 2017; Lopes et al., 2018).

This question of differences between populations is definitely relevant. While we can assume some similarities and differences based on the literature, it will be necessary to lead the same approach with patients and clinicians in order to confront the results, deepen our knowledge and increase our ability to adapt BCIs accordingly. Once more, this will be a lever to improve BCI efficiency. Beyond the differences depending on the status of the respondents (patients, clinicians, and general public), there might also be differences related to their culture (Straub et al., 1997). Therefore, it also seems necessary to apply this approach on different populations around the world.

Collecting more data on diverse populations will enable us to refine our model. It is classic for acceptability models to evolve and to be adjusted to the time and context. The two versions of the UTAUT give us a perfect illustration of the necessity of adaptations. Indeed, whereas the first one was rather adapted to technologies for organizations (Venkatesh et al., 2003), the second one gravitates toward individual consumers/users (Venkatesh et al., 2012). For appropriate adaptations to be made, an open science approach will be necessary. Indeed we think that it will be possible only if people collect data, share their findings and work together on improving the soundness and reliability of the model.

###  4.1. Recommendations

These results offer first trails to make BCI-based stroke rehabilitation procedures more acceptable. On the one hand, we have seen that the video which explains the use of BCIs in post-stroke rehabilitation had an influence on BI and PU scores and on the predictors of these scores. Thus, informing (future) users is a key step: it is necessary to be as clear as possible on the objectives of using a BCI, on its functioning, on the expected results, but also on the constraints related to the use (learning time, cognitive cost, etc.). These recommendations are important to consider to improve the perception of the *benefits/risk ratio* and *relevance* factors. We think that one of the most interesting formats of information can be the production of educational videos that help demystify BCIs, as we did in the questionnaire. This is in line with what could be done to take ***social*** ***influence*** into account. Indeed, one of the best ways to play on *subjective norm*, and use the influence of this factor to improve acceptability, is to lead pedagogical actions on the general population. If people surrounding post-stroke subjects have an enlightened point of view on BCI, this could positively influence the acceptability of the therapy of this population. We want to underline that, to our viewpoint, these recommendations can be replicated in others BCIs settings, not only in post-stroke rehabilitation context.

Our most important recommendation, in any context of use of BCIs, remains to ensure that acceptability is assessed in order to adapt the protocol accordingly. It is an easy way to improve patients' well-being during rehabilitation phases and thereby, most certainly, to increase their engagement and thereby leverage the efficiency of BCI-based rehabilitation procedures.

## Conclusion

This paper is dedicated to the general public acceptability of BCI-based post-stroke rehabilitation procedures. We are conscious that collecting the opinions of post-stroke subjects and caregivers is also essential. We are currently working on this, conducting questionnaires and semi-structured interviews with post-stroke subjects and caregivers. This will allow us to investigate whether the acceptability factors that stand out the most are similar to those of the general public, and if not, to try to understand what could be the cause of these differences and how to move toward more personalized acceptability models, adapted to the targeted users.

## Data availability statement

The raw data supporting the conclusions of this article will be made available by the authors upon request, without undue reservation.

## Ethics statement

The studies involving human participants were reviewed and approved by Institutional Review Board of Toulouse Federal University (N°2019-140). The patients/participants provided their written informed consent to participate in this study.

## Author contributions

CJ-K, EG, KF, ST, FA, DG, JP, and LB conceived and designed the experiments. EG, KF, ST, and CJ-K performed the survey and analyzed the data. EG wrote the first manuscript. All authors contributed to the article and approved the submitted version.
