# Correction: Modeling the acceptability of BCIs for motor rehabilitation after stroke: a large scale study on the general public

**DOI:** 10.3389/fnrgo.2026.1854498

**Published:** 2026-07-06

**Authors:** Elise Grevet, Killyam Forge, Sebastien Tadiello, Margaux Izac, Franck Amadieu, Lionel Brunel, Léa Pillette, Jacques Py, David Gasq, Camille Jeunet-Kelway

**Affiliations:** 1 CNRS, EPHE, INCIA, UMR5287, Université de Bordeaux, Bordeaux, France; 2 CLLE, Université de Toulouse, CNRS, Toulouse, France; 3 Université Paul Valéry Montpellier 3, EPSYLON EA 4556, Montpellier, France; 4 ToNIC, Université de Toulouse, INSERM, Toulouse, France; 5 Centre Hospitalier Universitaire Toulouse, Toulouse, France

**Keywords:** brain-computer interface (BCI), neurofeedback (NF), acceptability, acceptance, stroke, motor rehabilitation, model, questionnaire

In the published article, there was an error in the values presented in Table 4. The corrected Table 4 appears below.

**Table 4:** Results from general public questionnaire.

Factor	Type	Mean	SD
System characteristics			
Result demonstrability	Quantitative	6.84	1.68
Benefits/risks ratio	Quantitative	7.27	1.51
Relevance	Quantitative	8.03	1.48
Image	Quantitative	6.10	2.17
Visual aesthetic	Quantitative	6.62	1.89
Social influence			
Subjective norm	Quantitative	7.38	1.71
Individual differences			
Autonomy	Quantitative	7.40	1.46
General anxiety	Quantitative	5.49	1.87
Computer anxiety	Quantitative	6.34	2.50
		**Condition**	**%**
Computer self-efficacy	Qualitative	Alone. independently	23.11%
*Usage conditions if BCI installed and explained*		Alone. if has experience with a similar technology	11.95%
		Alone. with support from a virtual companion	36.65%
		Only with human guidance and presence	28.29%
Social support	Qualitative	Independently. alone at home	32.80%
		In the presence of a health professional	47.70%
		Alone. but in a health facility	19.50%
BCI Knowledge	Qualitative	Yes-has already used a BCI	3.98%
		Yes-but has never used a BCI	27.09%
		No	68.66%
		**Mean**	**SD**
Facilitating conditions			
Agency	Quantitative	6.29	1.65
Playfulness	Quantitative	6.90	1.79
Ease of learning	Quantitative	5.96	1.62
Characteristics of the system			
Result demonstrability	Quantitative	6.84	1.68
Benefits/risks ratio	Quantitative	7.27	1.51
Relevance	Quantitative	8.03	1.48
Image	Quantitative	6.10	2.17
Visual aesthetic	Quantitative	6.62	1.89
Main target factors			
BI	Quantitative	7.87	1.72
PU	Quantitative	7.87	1.63
PEOU	Quantitative	7.17	1.57
PU 2	Quantitative	8.28	1.57
BI 2	Quantitative	8.21	1.67

Scores for the quantitative variables were continuous from 0 to 10 (*strongly disagree* to *strongly agree*).

In the published article, there was an error in the values presented in Table 6. The corrected Table 6 appears below.

**Table 6:** Table of scores for mediation analysis (only with quantitative factors).

Category	Independent variable (IV)	Total effect	Effect IV-MV		Effect MV-DV		Direct effect	Indirect effect	BC 95% CI of indirect effect (bootstrap: nb iterations = 500)
			PU2	PEOU	PU2	PEOU			
System Characteristics - PEOU	Visual aesthetic	0.32^**^	–	0.38^**^	–		0.04	0.28	[0.22; 0.34]
		*p*= 3.19e-22		*p* = 2.48e-43		0.72^**^	*p* = 1.47e-01		
	Image	0.09	–	0.17^**^		*p* = 3.29e-76	−0.03	0.12	[0.22; 0.5]
		*p* = 8.97e-04		*p* = 8.81e-14			*p* = 1.26e-01		
System characteristics-PU2	Relevance	0.54^**^	0.65^**^	–		–	0.04	0.50	[0.41; 0.60]
		*p* = 6.24e-51	*p* = 6.24e-83				*p* = 1.78e-01		
	PEOU	0.19^**^	0.2^**^	–			0.04	0.15	[0.09; 0.21]
		*p* = 2.67e-11	*p* = 2.77e-14				*p* = 5.17e-02		
	Benefits/Risks ratio	0.23^**^	0.15^**^	–	0.77^**^		0.11^**^	0.12	[0.06; 0.19]
		*p* = 1.73e-13	*p* = 1.51e-08		*p* = 1.08e-102		*p* = 4.12e-06		
	Result demonstrability	0.14^**^	0.04	–			0.11^**^	0.03	[0.0; 0.07]
		*p* = 1.73e-08	*p* = 5.15e-02				*p* = 4.18e-09		
	Visual aesthetic	0	−0.02	–			0.02	−0.01	[-0.04; 0.01]
		*p* = 8.34e-01	*p* = 3.06e-02				*p* = 2.17e-01		
	Image	−0.02	−0.01	–			−0.01	−0.01	[-0.03; 0.01]
		*p* = 2.76e-01	*p* = 5.26e-01				*p* = 3.70e-01		
Social influence	Subjective norm	0.63^**^	0.58^**^	0.57^**^	0.89^**^	0.06	0.08^**^	0.55	[0.47; 0.61]
		*p* = 2.48e-91	*p* = 5.68e-86	*p* = 1.88e-80	*p* = 4.95e-198	*p* = 6.04e-03	*p* = 2.16e-06		
Individual differences	Autonomy	0.34^**^	0.36^**^	0.33^**^			−0.01	0.35	[0.27; 0.42]
		*p* = 1.32e-19	*p* = 1.24e-23	*p* = 8.39e-19			*p* = 4.85e-01		
	Computer anxiety	0.27^**^	0.22^**^	0.15^**^	0.89^**^	0.09^**^	0.06^**^	0.21	[0.16; 0.26]
		*p* = 4.42e-33	*p* = 4.94e-26	*p* = 1.68e-12	*p* = 1.17e-202	*p* = 5.87e-06	*p* = 2.27e-10		
	General anxiety	−0.05	−0.04	−0.04			−0.01	−0.04	[−0.09; 0.02]
		*p* = 1.20e-01	*p* = 1.43e-01	*p* = 1.34e-01			*p* = 6.74e-01		
Facilitating conditions	Playfulness	0.41^**^	0.36^**^	0.39^**^		0.01 *p* = 5.50e-01	0.09^**^	0.32	[0.25; 0.39]
		*p* = 1.69e-25	*p* = 3.25e-21	*p* = 1.38e-30			*p* = 3.09e-06		
	Agency	0.28^**^	0.25^**^	0.06	0.88^**^		0.05	0.22	[0.16; 0.29]
		*p* = 3.76e-13	*p* = 8.75e-12	*p* = 5.87e-02	*p* = 4.12e-203		*p* = 2.88e-03		
	Ease of learning	0	−0.01	0.29			0.01	−0.01	[−0.08; 0.06]
		*p* = 9.64e-01			*p* = 6.90e-01	*p* = 6.19e-19			*p* = 5.78e-01
Main target factors	PEOU	0.73^**^	0.69^**^	–	0.92^**^	–	0.09^**^	0.64	[0.58; 0.71]
		*p* = 9.94e-105	*p* = 4.25e-110		*p* = 1.02e-220		*p* = 1.03e-05				

The mediators variable were PU2 and PEOU, the dependant variable was BI2. ^**^p < 0.001–BC, bias corrected; DV, dependent variable; MV, mediator variable; PU, perceived usefulness; PEOU, perceived ease of use. Color differences are for ease of reading the table, no special meaning.

In the published article, there was an error in the values reported in Figure 4. Instead of “0.94” and “0.08,” the values should have been “0.92” and 0.09.” The corrected Figure 4 appears below.

**Figure 4:**
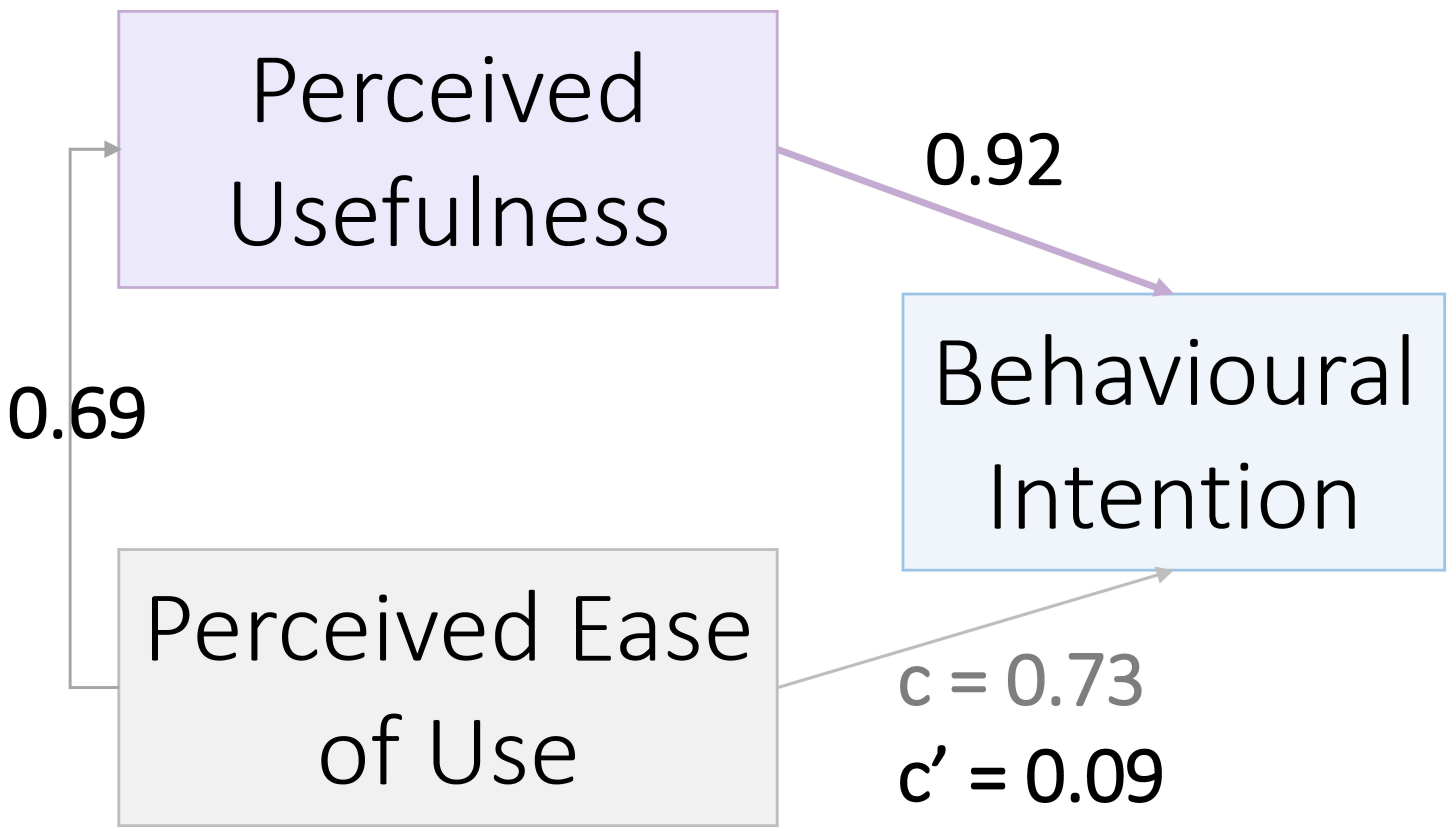
Mediation analysis for the target factors: Behavioral intention (BI2), Perceived usefulness (PU2) and Perceived ease of use (PEOU). *R*^2^ = 0.86 (*p* < 0.001). c, total effect of PEOU on BI2; c', direct effect of PEOU on BI2; c-c', indirect effect of PEOU on BI2 through PU2.

In the published article, there was an error in the values presented for BI2 in Table 8. Instead of “0.63,” “0.39,” “0.64,” “0.71,” and “0.74,” the values should have been “0.65,” “0.40,” “0.65,” “0.73,” and “0.75.” The corrected Table 8 appears below.

**Table 8:** Spearman correlation analyzes for the most significant factors.

Target factors	Subjective norm	Visual aesthetics	Playfulness	Ease of learning	PEOU	Result demonstrability	Benefits/risks ratio	Relevance	PU2
PEOU	0.59	0.54	0.63	0.6					
	*p* < 0.001	*p* < 0.001	*p* < 0.001	*p* < 0.001					
PU2	0.63	0.39	0.55	0.37	0.66	0.69	0.74	0.89	
	*p* < 0.001	*p* < 0.001	*p* < 0.001	*p* < 0.001	*p* < 0.001	*p* < 0.001	*p* < 0.001	*p* < 0.001	
BI2	0.65	0.41	0.59	0.40	0.65	0.73	0.75	0.86	0.92
	*p* < 0.001	*p* < 0.001	*p* < 0.001	*p* < 0.001	*p* < 0.001	*p* < 0.001	*p* < 0.001	*p* < 0.001	*p* < 0.001

*p*-values adjusted with Bonferroni correction. The color shade is intended to show the value of the coefficient. The closer a coefficient is to 1, the more the color is intense.

In the published article, in the Section *3.4.1. Important factors in each category of our model: Mediation analysis*, paragraph 2, there was an error in the results as reported. The sentence: “Figure 4 shows that the BI was mainly influenced by PU2 (effect: 0.94, p < 0.001), the weight of the PEOU being much lower (direct effect: 0.08, standard error SE = 0.02, p < 0.001; indirect effect: 0.65, SE = 0.03, CI = [0.59, 0.71]), i.e., PEOU had a low effect on BI2 but a significant effect on PU2.” Has been corrected to: “Figure 4 shows that the BI was mainly influenced by PU2 (effect: 0.92, *p* < 0.001), the weight of the PEOU being much lower [direct effect: 0.09, standard error SE = 0.02, *p* < 0.001; indirect effect: 0.64, SE = 0.03, CI = [0.58, 0.71]], i.e., PEOU had a low effect on BI2 but a significant effect on PU2.”

In the published article, in the Section *3.2.2. Descriptive analysis*, paragraph 1, there was an error in the BI2 mean score as reported. The sentence: “Indeed, regarding the target factors, BI2 had a mean of 8.23 (*SD* = 1.69)” has been corrected to: “Indeed, regarding the target factors, BI2 had a mean of 8.21 (*SD* = 1.67).”

In the published article, in the Section *3.2.2. Descriptive analysis*, paragraph 3, there was an error in the BI2 mean score as reported. The sentence: “Before video 2: PU1 mean = 7.87, SD = 1.63/BI1 mean = 7.88, SD = 1.73. After video 2: PU2 mean = 8.28, SD = 1.57/BI2 mean = 8.23, SD = 1.69.” has been corrected to: “Before video 2: PU1 mean = 7.87, SD = 1.63/BI1 mean = 7.88, SD = 1.73. After video 2: PU2 mean = 8.28, SD = 1.57/BI2 mean = 8.21, SD = 1.67.”

In the published article, in the **4. Discussion** Section, paragraph 5, there was an error in the BI2 score as reported. The sentence: “After the second video, participants globally increased their rating and gave PU2 scores of 8.28+/1.57, and BI2 scores of 8.23+/−1.69.” has been corrected to: “After the second video, participants globally increased their rating and gave PU2 scores of 8.28+/1.57, and BI2 scores of 8.21 ± 1.67.”

In the published article, in the Section *3.4.1. Important factors in each category of our model: Mediation analysis*, paragraphs 3–6, the following results contained significant errors:

“Concerning the other categories of our model, our results revealed that for the individual differences, autonomy was the most influential factor on BI, but this effect was moderate (c = 0.34, *p* < 0.001), it equally impacted PU2 and PEOU (respectively, 0.36 and 0.33, with *p* < 0.001) (quality of the model: *R*^2^ = 0.87, *p* = 0.0).

For social influence, subjective norm had a similar and moderate impact on both PU2 and PEOU (respectively, 0.58 and 0.57, with *p* < 0.001). The influence on BI2 was rather high (c = 0.63, *p* < 0.001) (quality of the model: *R*^2^ = 0.86, *p* < 0.001).

For characteristics of the system, we did two analyse: (i) one with only PEOU as mediator, and factors present before video 2 (PU1 was not included since we chose to focus on PU2); (ii) the second only with PU2 as mediator, and factors present before and after video 2 (PEOU was among these factors since, as shown in Figure 2, it influences PU). (i) shows that visual aesthetics was the most—but weak—influential factor on PEOU (0.38, with p < 0.001). The total effect of visual aesthetics on BI2 was low: C = 0.32 (*p* < 0.001) (quality of the model: *R*^2^ = 0.47, *p* < 0.001). On the other hand (ii) revealed that relevance was the most influential factor on PU2 (0.65, with *p* < 0.001). Its total effect on BI2 was C = 0.56 (*p* < 0.001) (quality of the model: *R*^2^ = 0.87, *p* = 0.0).

Finally, for facilitating conditions, the variable with most impact was computer playfulness, it equally impacted PU2 and PEOU (respectively, 0.36 and 0.39, with *p* < 0.001). The influence of computer playfulness on BI2 was moderate (C = 0.41, *p* < 0.001) (quality of the model: *R*^2^ = 0.86, *p* < 0.001). Additional figures of mediation analysis are disponible in **Supplementary material 2**.”

These paragraphs have now been corrected to:

“Concerning the other categories of our model, our results revealed that for the *individual differences, autonomy* was the most influential factor on BI, but this effect was moderate (c = 0.34, *p* < 0.001), it equally impacted PU2 and PEOU (respectively, 0.36 and 0.33, with *p* < 0.001) (quality of the model: *R*^2^ = 0.87, *p* < 0.001).

For *social influence, subjective norm* had a similar and moderate impact on both PU2 and PEOU (respectively, 0.58 and 0.57, with *p* < 0.001). The influence on BI2 was rather high (c = 0.63, *p* < 0.001) (quality of the model: *R*^2^ = 0.87, *p* < 0.001).

For *characteristics of the system*, we did two analyses: (i) one with only PEOU as mediator, and factors present before video 2 (PU1 was not included since we chose to focus on PU2); (ii) the second only with PU2 as mediator, and factors present before and after video 2 (PEOU was among these factors since, as shown in Figure 2, it influences PU). **(i)** shows that *visual aesthetics* was the most—but weak—influential factor on PEOU (0.38, with p < 0.001). The total effect of *visual aesthetics* on BI2 was low: c = 0.32 (*p* < 0.001) (quality of the model: *R*^2^ = 0.48, *p* < 0.001). On the other hand, (ii) revealed that *relevance* was the most influential factor on PU2 (0.65, with *p* < 0.001). Its total effect on BI2 was c = 0.54 (*p* < 0.001) (quality of the model: *R*^2^ = 0.88, *p* < 0.001).

Finally, for *facilitating conditions*, the variable with most impact was *computer playfulness*, it equally impacted PU2 and PEOU (respectively, 0.36 and 0.39, with *p* < 0.001). The influence of *computer playfulness* on BI2 was moderate (c = 0.41, *p* < 0.001) (quality of the model: *R*^2^ = 0.87, *p* < 0.001). Additional figures of mediation analysis are available in **Supplementary material 2**.”

In the published article, in the **4. Discussion** Section, paragraph 3, there was an error in the reported average acceptability level score. The sentence: “A main result of this study is that, globally, acceptability levels in terms of behavioral intention seem to be very high in the general public (with an average score of 8.23/10).” has been corrected to: “A main result of this study is that, globally, acceptability levels in terms of behavioral intention seem to be very high in the general public (with an average score of 8.21/10).”

The original version of this article has been updated.

