# Supplementary material for: Modeling the acceptability of BCIs for motor rehabilitation after stroke: A large scale study on the general public
Source: Front Neuroergon. 2023 Feb 1;3:1082901. doi: 10.3389/fnrgo.2022.1082901 (PMC10790937; doi:10.3389/fnrgo.2022.1082901)
Supplement: Supplementary file 1 [file Data_Sheet_1.pdf]

Modelling the acceptability of BCIs for motor rehabilitation after stroke: a large scale study on the general public

Elise Grevet, Killyam Forge, Sebastien Tadiello, Margaux Izac, Franck Amadieu, Lionel Brunel, Léa Pillette, Jacques Py, David Gasq and Camille Jeunet-Kelway
Correspondence*: Elise Grevet - elise.grevet@u-bordeaux.fr

Caution : it is possible that the English version has wording errors, we propose it here to facilitate the understanding of non-French readers, but it has not yet been validated.

Facteur mesuré	Questions - French	Measured factor	Questions - English
Plaisir face aux technologies	Je prends plaisir à utiliser les nouvelles technologies.	Pleasure towards the use of technologies	I enjoy using new technologies.
Confiance face aux technologies	En règle générale, je me sens en confiance lorsque j'interagis avec des nouvelles technologies.	Confidence towards the use of technologies	Generally, I feel confident when interacting with new technologies.
Autonomie	J'apprécie plus le fait d'accomplir des choses que de recevoir une récompense en échange.	Autonomy	I enjoy accomplishing things more than being given credit for them.
Autonomie	Il est important pour moi d'être libre et indépendant(e).	Autonomy	It is very important that I feel free to get up and go where ever I want.
Autonomie	Je ne suis pas influencé(e) par les autres dans ce que je décide de faire.	Autonomy	It is more important to meet your own objectives on a task than to meet another person's objective.
Anxiété générale	De manière générale, je me sens nerveux (nerveuse) et agité(e).	General anxiety	In general, I feel nervous and restless.
Anxiété générale	Je m'inquiète souvent à propos de choses sans importance.	General anxiety	I worry too much over something that really doesn't matter.
Anxiété générale	En règle générale, je me sens heureux (heureuse).	General anxiety	In general, I am happy.
Anxiété générale	Je me sens la plupart du temps sans inquiétude, en sécurité, en sûreté.	General anxiety	In general, I am "calm, cool, and collected".
Anxiété générale	Je deviens tendu(e) et agité(e) quand je réfléchis à mes soucis.	General anxiety	I get in state of tension or turmoil as I think over my recent concerns and interests.
Normes subjectives	Je pense que le grand public serait assez ouvert à l'utilisation de tels systèmes.	Subjectives norm	I think the general public would support the use of such systems.
Normes subjectives	Les personnes qui sont importantes pour moi seraient favorables à l'utilisation des Interfaces Cerveau-Ordinateur dans le cadre d'une rééducation post-AVC.	Subjectives norm	People who are important to me would be in favour of brain-computer interfaces in post-stroke motor rehabilitation.
Normes subjectives	Les personnes dont l'avis m'importe seraient favorables à l'utilisation des Interfaces Cerveau-Ordinateur dans le cadre d'une rééducation post-AVC.	Subjectives norm	People who influence my behavior would think that I should use brain-computer interfaces in post-stroke motor rehabilitation.
Image	Les patients en rééducation qui accepteront d'utiliser une Interface Cerveau-Ordinateur pourraient être perçus plus positivement que ceux qui refuseront.	Image	People who will accept to use brain-computer interfaces in post-stroke motor rehabilitation would have more prestige than those who will not.
Image	Les patients qui accepteront d'utiliser une Interface Cerveau-Ordinateur dans le cadre de leur rééducation post-AVC renverront une meilleure image d'eux mêmes que ceux qui refuseront.	Image	People who will using brain-computer interfaces in post-stroke motor rehabilitation would have a higher profile than whose who will not.
Image	Accepter d'utiliser une Interfaces Cerveau-Ordinateur suite à un AVC pourrait refléter un statut social plus élevé que celui des personnes qui s'y refuseront.	Image	Using a brain-computer interface in post-stroke motor rehabilitation would be a symbol of status.
Anxiété face à l'ordinateur	Utiliser une Interface Cerveau-Ordinateur me rendrait nerveux/nerveuse.	Computer anxiety	Using a brain-computer interface would make me nervous.
Anxiété face à l'ordinateur	L'utilisation d'Interfaces Cerveau-Ordinateur me mettrait mal à l'aise.	Computer anxiety	Brain-computer interfaces would make me feel uncomfortable.
Anxiété face à l'ordinateur	L'idée d'utiliser une Interface Cerveau-Ordinateur me rend anxieux/anxieuse.	Computer anxiety	Brain-computer interfaces would make me feel anxious.
Esthétique	Le design des Interfaces Cerveau-Ordinateur, en termes de matériel et d'affichages à l'écran, a l'air attrayant.	Visual aesthetics	The design of brain-computer interfaces, in terms of materials and screen displays, looks appealing.
Esthétique	L'esthétique des Interfaces Cerveau-Ordinateur a l'air plaisante.	Visual aesthetics	The aesthetics of brain-computer interfaces seems to be pleasant.
Esthétique	Le design des Interfaces Cerveau-Ordinateur, en termes de matériel et d'affichages à l'écran, a l'air bien pensé.	Visual aesthetics	The design of brain-computer interfaces, in terms of materials and screen displays, looks well designed.
Agentivité	Si j'utilisais une Interface Cerveau-Ordinateur, je pense que je me sentirais agent, en contrôle.	Agency	I think I would have control over using brain-computer interfaces.
Agentivité	Si j'utilisais une Interface Cerveau-Ordinateur, je n'aurais pas l'impression de contrôler ce qui se passe.	Agency	If I was using a brain-computer interface, I would feel like I am not in control of what is going on.
Agentivité	Si j'utilisais une Interface Cerveau-Ordinateur, je pense que je me sentirais maître(sse) de mes actions.	Agency	If I was using a brain-computer interface, I think I would feel in control of my actions.
Aspect ludique	Je trouve que les Interfaces Cerveau-Ordinateur ont l'air agréables à utiliser.	Playfulness	I find using brain-computer interfaces seems to be enjoyable.
Aspect ludique	La rééducation avec ce genre de système a l'air plaisante.	Playfulness	Using brain-computer interfaces in rehabilitation seems pleasant.
Aspect ludique	Je pense que je trouverais cela amusant d'utiliser une Interface Cerveau-Ordinateur.	Playfulness	I think I would have fun during the use of a brain-computer interface.
Facilité d'apprentissage	Je pense qu'il est facile d'apprendre à se servir d'une Interface Cerveau-Ordinateur.	Ease of learning	I think it would be easy to learn to use a brain-computer interface.
Facilité d'apprentissage	J'ai l'impression qu'apprendre à me servir d'une Interface Cerveau-Ordinateur me demanderait beaucoup d'énergie.	Ease of learning	I think that learning how to use a brain-computer interface would take me a lot of energy.
Facilité d'apprentissage	Je pense que les gens pourraient rapidement apprendre à utiliser une Interface Cerveau-Ordinateur.	Ease of learning	I think people would quickly became skillful with brain-computer interface.
Utilité perçue 1	Selon moi, utiliser une Interface Cerveau-Ordinateur permettrait réellement d'améliorer la récupération motrice.	Perceived usefulness (PU1)	In my opinion, using a brain-computer interface would really improve motor recovery.
Utilité perçue 1	Je pense qu'utiliser une Interface Cerveau-Ordinateur suite à un AVC rendrait la rééducation des patients plus efficace et plus rapide.	Perceived usefulness (PU1)	I think that using a brain-computer interface in post-stroke would make the rehabilitation of patients more efficient and faster.
Utilité perçue 1	Selon moi, utiliser une Interface Cerveau-Ordinateur serait utile à la rééducation après un AVC.	Perceived usefulness (PU1)	In my opinion, using a brain-computer interface would be useful for motor recovery
Facilité d'usage perçue	Il me semble qu'utiliser une Interface Cerveau-Ordinateur ne nécessiterait pas d'efforts particuliers.	Perceived ease of use (PEOU)	I think becoming skillful in brain-computer interface use would not require special efforts.
Facilité d'usage perçue	Selon moi, les Interfaces Cerveau-Ordinateur seraient faciles à utiliser.	Perceived ease of use (PEOU)	I think using a brain-computer interface would be easy.
Facilité d'usage perçue	Je pense qu'en général, les gens pourraient facilement apprendre à utiliser une Interface Cerveau-Ordinateur.	Perceived ease of use (PEOU)	I think people could easily learn to use a brain-computer interface.
Intention d'usage 1	Si quelqu'un en avait l'opportunité, je lui conseillerais d'utiliser une Interface Cerveau-Ordinateur dans le cadre de sa rééducation.	Behavioural intention (BI1)	If someone had the opportunity, I would advise him to use a brain-computer interface in his/her post-stroke rehabilitation.
Intention d'usage 1	Si j'avais la possiblité d'utiliser une Interface Cerveau-Ordinateur dans le cadre d'une rééducation, je le ferais.	Behavioural intention (BI1)	Assuming I had access to a brain-computer interface for rehabilitation, I would intend to use it.
Intention d'usage 1	Si je devais effectuer une rééducation, je souhaiterais utiliser une Interface Cerveau-Ordinateur.	Behavioural intention (BI1)	If I had to do rehabilitation, I would want to use a brain-computer interface.
Intention d'usage 1	Si un proche en avait la possibilité, je lui conseillerais d'utiliser une Interface Cerveau-Ordinateur.	Behavioural intention (BI1)	If a close relative had this possibility, I would advise him to use a brain-computer interface.
Démonstrabilité des résultats	Je n'aurais aucune difficulté à expliquer à d'autres personnes l'intérêt d'utiliser une Interface Cerveau-Ordinateur.	Result demonstrability	I would have no trouble to explain to others the value of using a brain-computer interface.
Démonstrabilité des résultats	Je crois que je pourrais communiquer à d'autres personnes les conséquences potentielles de l'utilisation de tels systèmes.	Result demonstrability	I believe I could communicate to others the consequences of using a brain-computer interface.
Démonstrabilité des résultats	Les résultats attendus suite à l'utilisation d'une Interface Cerveau-Ordinateur me semblent clairs.	Result demonstrability	The expected results of using a brain-computer interface are apparent to me.
Démonstrabilité des résultats	J'aurais du mal à expliquer en quoi l'utilisation d'un tel système pourrait ou non être bénéfique.	Result demonstrability	I would struggle to explain why using a brain-computer interface may or not be beneficial.
Ratio bénéfices/risques	Je pense qu'utiliser une Interfaces Cerveau-Ordinateur représenterait un danger pour moi.	Benefits/risk ratio	I think using a brain-computer interface would be dangerous for me.
Ratio bénéfices/risques	Les Interfaces Cerveau-Ordinateur semblent n'avoir que des avantages.	Benefits/risk ratio	Brain-computer interfaces seem to have only advantages.
Ratio bénéfices/risques	Les Interfaces Cerveau-Ordinateur semblent peu contraignantes.	Benefits/risk ratio	Brain-computer interfaces seem not too restrictive.
Ratio bénéfices/risques	Je pense que les bénéfices liés à l'usage d'Interfaces Cerveau-Ordinateur sont supérieurs aux risques.	Benefits/risk ratio	I think the benefits of using brain-computer interfaces outweigh the risks.
Pertinence	Il semblerait qu'utiliser une Interface Cerveau-Ordinateur soit pertinent pour la rééducation post-AVC.	Relevance	To post-stroke motor rehabilitation, the use of a brain-computer interface would be relevant.
Pertinence	Les scientifiques suggèrent que l'utilisation des Interfaces Cerveau-Ordinateur serait importante pour retrouver une autonomie dans les tâches du quotidien.	Relevance	Scientists suggest that the use of brain-computer interfaces would be important to regain autonomy in daily tasks.
Pertinence	L'utilisation d'Interfaces Cerveau-Ordinateur pourrait rendre la rééducation plus efficace et plus rapide.	Relevance	The use of brain-computer interfaces could enable a more efficiently and faster rehabilitation.
Pertinence	Les avancées dans le domaine indiquent que l'utilisation des Interfaces Cerveau-Ordinateur serait plus pertinente qu'une rééducation classique.	Relevance	Advances in the field indicate that the use of brain-computer interfaces would be more relevant than a classic rehabilitation.
Utilité perçue 2	Selon moi, utiliser une Interface Cerveau-Ordinateur permettrait réellement d'améliorer la récupération motrice.	Perceived usefulness (PU2)	In my opinion, using a brain-computer interface would really improve motor recovery.
Utilité perçue 2	Je pense qu'utiliser une Interface Cerveau-Ordinateur suite à un AVC rendrait la rééducation des patients plus efficace et plus rapide.	Perceived usefulness (PU2)	I think that using a brain-computer interface in post-stroke would make the rehabilitation of patients more efficient and faster.
Utilité perçue 2	Selon moi, utiliser une Interface Cerveau-Ordinateur serait utile à la rééducation après un AVC.	Perceived usefulness (PU2)	In my opinion, using a brain-computer interface would be useful for motor recovery
Intention d'usage 2	Si quelqu'un en avait l'opportunité, je lui conseillerais d'utiliser une Interface Cerveau-Ordinateur dans le cadre de sa rééducation.	Behavioural intention (BI2)	If someone had the opportunity, I would advise him to use a brain-computer interface in his/her post-stroke rehabilitation.
Intention d'usage 2	Si j'avais la possiblité d'utiliser une Interface Cerveau-Ordinateur dans le cadre d'une rééducation, je le ferais.	Behavioural intention (BI2)	Assuming I had access to a brain-computer interface for rehabilitation, I would intend to use it.
Intention d'usage 2	Si je devais effectuer une rééducation, je souhaiterais utiliser une Interface Cerveau-Ordinateur.	Behavioural intention (BI2)	If I had to do rehabilitation, I would want to use a brain-computer interface.
Intention d'usage 2	Si un proche en avait la possibilité, je lui conseillerais d'utiliser une Interface Cerveau-Ordinateur.	Behavioural intention (BI2)	If a close relative had this possibility, I would advise him to use a brain-computer interface.
Support social	Je préfèrerais utiliser une Interface Cerveau-Ordinateur :<br>- En autonomie, seul(e) chez moi.<br>- Seul(e), mais dans un établissement de santé.<br>- En présence d'un(e) professionnel(le) de santé.	Social support	I would prefer to use a brain-computer interface:<br>- In autonomy, alone at home.<br>- Alone, but in a health facility.<br>- In the presence of a health professional.
Auto-efficacité	A condition qu'on m'installe le système et qu'on m'explique comment il fonctionne, je pense que je serais capable d'utiliser une Interface Cerveau-Ordinateur :<br>- Seul(e), en autonomie<br>-Seul(e), à condition de disposer simplement d'une fonction de support intégrée à l'interface pour obtenir de l'aide, par exemple sous forme de compagnon virtuel.<br>- Seul(e), à condition d'avoir déjà utilisé une technologie similaire auparavant.<br>-Uniquement si quelqu'un me montrait comment effectuer la tâche et me guidait au fur et à mesure.	Self-efficacy	Provided that the system is installed and that someone explains to me how it works, I think I would be able to use a brain-computer interface:<br>- Alone, independently<br>- Alone, provided I simply have a support function integrated into the interface to obtain help, for example in the form of a virtual companion.<br>- Alone, provided I have already used a similar technology before.<br>-Only if someone showed me how to do the task and guide me bit by bit.
Connaissance des BCIs et du neurofe	Avez-vous déjà entendu parler d'Interfaces Cerveau-Ordinateur et/ou de Neurofeedback ?	BCI and NF knowledge	Have you ever heard of brain-computer interfaces and/or neurofeedback?
Rapport à l'AVC	Avez-vous déjà eu un AVC ?	Stroke	Have you ever had a stroke?
Rapport à l'AVC	Êtes-vous actuellement hospitalisé(e) suite à votre AVC ?	Stroke	Are you currently hospitalised following your stroke?
Rapport à l'AVC<br>*si sujet n'a jamais eu d'AVC*	Avez-vous une ou plusieurs personnes de votre entourage proche (famille, amis) qui ont eu un AVC ?	Stroke<br>*if subject has never had a stroke*	Do you have one or more people in your close circle (family, friends) who have had a stroke?
Rapport à l'AVC<br>*si sujet n'a jamais eu d'AVC*	A quelle fréquence êtes vous impliqué(e) dans la rééducation de ce(s) proche(s) ayant eu un AVC ?	Stroke<br>*if subject has never had a stroke*	How often are you involved in the rehabilitation of these close relations who have had a stroke?
Utilisation de technologies	Parmi ces technologies, laquelle/lesquelles utilisez-vous régulièrement, c'est-à-dire plusieurs fois par mois ?<br>- Smartphone / Montre connectée / Assistant à commande vocale / Assistant à commande vocale (type Google Home, Alexa…) / Outils de réalité augmentée / Outils de réalité virtuelle / Véhicule autonome<br>- Je n'utilise aucune de ces technologies régulièrement (seulement occasionnellement)<br>- Je n'utilise jamais ces technologies	Technologies usage	Which of these technologies do you use regularly, i.e. several times a month?<br>- Smartphone / Connected watch / Voice-activated assistant (like Google Home, Alexa, etc.) / Augmented reality tools / Virtual reality tools / Autonomous vehicle<br>- I don't use any of these technologies regularly (only occasionally)<br>- I never use these technologies
Genre	Vous identifiez-vous comme : Une femme / Un homme / Autre / Je ne souhaite pas répondre	Gender	Do you identify as: A Woman / A Man / Other
Age	Quel âge avez-vous ?	Age	How old are you?
Diplôme	Quel est votre plus haut diplôme obtenu ?	Diploma	What is your highest degree obtained?
Catégorie socioprofessionnelle	Quelle est votre catégorie socio-professionnelle ? (si vous êtes à la retraite, indiquez celle lorsque vous étiez en activité)	Socio-professional category	What is your socio-professional category?
