# Supplementary material for: Modeling the acceptability of BCIs for motor rehabilitation after stroke: A large scale study on the general public
Source: Front Neuroergon. 2023 Feb 1;3:1082901. doi: 10.3389/fnrgo.2022.1082901 (PMC10790937; doi:10.3389/fnrgo.2022.1082901)
Supplement: Supplementary file 2 [file Data_Sheet_2.pdf]

Supplementary Material - Modelling the acceptabilityof BCIs for motor rehabilitation after stroke: a large scale study on the general public

1 CRONBACH'S ALPHA - RESULTS FROM R SOFTWARE

The factors were those after the second video, so they were all present excepted BI1 and PU1.

```
lavaan 0.6-9 ended normally after 180 iterations

  Estimator                                         ML
  Optimization method                           NLMINB
  Number of model parameters                       240

  Number of observations                           753

Model Test User Model:
                                              Standard      Robust
  Test Statistic                              4323.835    3346.576
  Degrees of freedom                              1191        1191
  P-value (Chi-square)                           0.000       0.000
  Scaling correction factor                                  1.292
       Yuan-Bentler correction (Mplus variant)

Model Test Baseline Model:

  Test statistic                             31078.101   22368.302
  Degrees of freedom                              1378        1378
  P-value                                        0.000       0.000
  Scaling correction factor                                  1.389

User Model versus Baseline Model:

  Comparative Fit Index (CFI)                    0.895       0.897
  Tucker-Lewis Index (TLI)                       0.878       0.881

  Robust Comparative Fit Index (CFI)                         0.905
  Robust Tucker-Lewis Index (TLI)                            0.890

Loglikelihood and Information Criteria:

  Loglikelihood user model (H0)             -73432.909  -73432.909
  Scaling correction factor                                  1.810
      for the MLR correction
  Loglikelihood unrestricted model (H1)     -71270.992  -71270.992
  Scaling correction factor                                  1.379
      for the MLR correction

  Akaike (AIC)                              147345.818  147345.818
  Bayesian (BIC)                            148455.594  148455.594
```

```
  Sample−size adjusted Bayesian (BIC)       147693.498   147693.498

Root Mean Square Error of Approximation:

  RMSEA                                         0.059        0.049
  90 Percent confidence interval − lower        0.057        0.047
  90 Percent confidence interval − upper        0.061        0.051
  P−value RMSEA $<$= 0.05                         0.000        0.822

  Robust RMSEA                                               0.056
  90 Percent confidence interval − lower                     0.054
  90 Percent confidence interval − upper                     0.058

Standardized Root Mean Square Residual:

  SRMR                                          0.078        0.078
```

2 MEDIATION ANALYSIS - DIAGRAMS OF THE ANALYSES FOR THE DIFFERENT CATEGORIES OF OUR MODEL

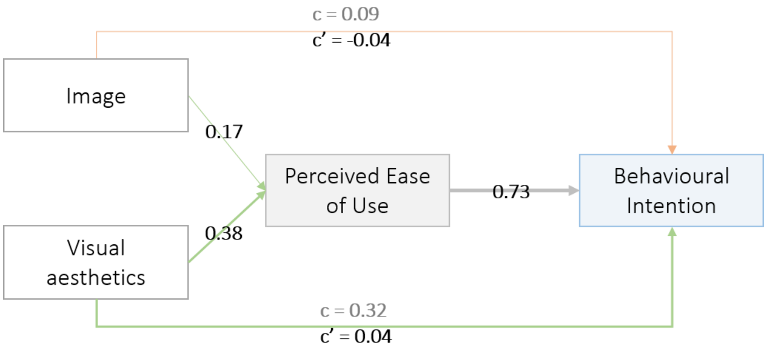

Figure S1. *PEOU* - Mediation analysis for the ***system characteristics***: *Visual aesthetics* is the most influential factor on PEOU (before the second explanatory video). R^2 = 0.47 (p-value < 0.001)

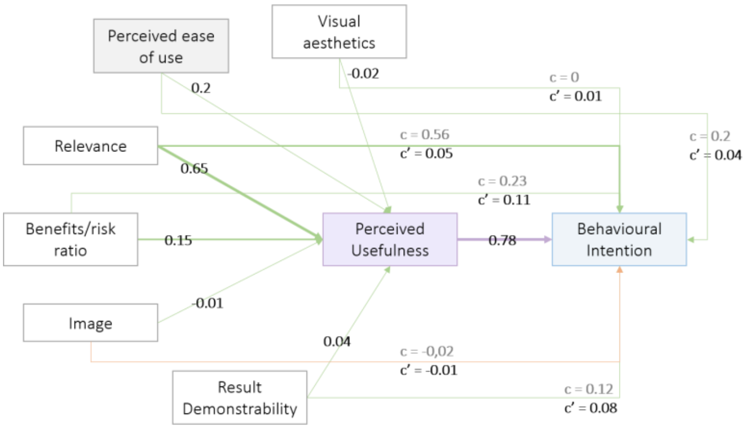

Figure S2. *PU2* - Mediation analysis for the ***system characteristics***: *Relevance* is the most influentialfactor on PU (after the second explanatory video). R^2 = 0.87 (p-value < 0.001)

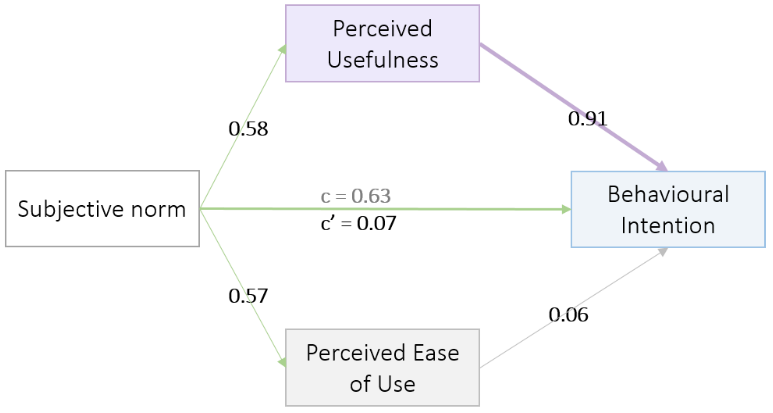

Figure S3. Mediation analysis for the ***social influence:*** *Subjective norm* has a similar and moderate impact on both PU and PEOU. $R^2 = 0.86$ (p-value < 0.001)

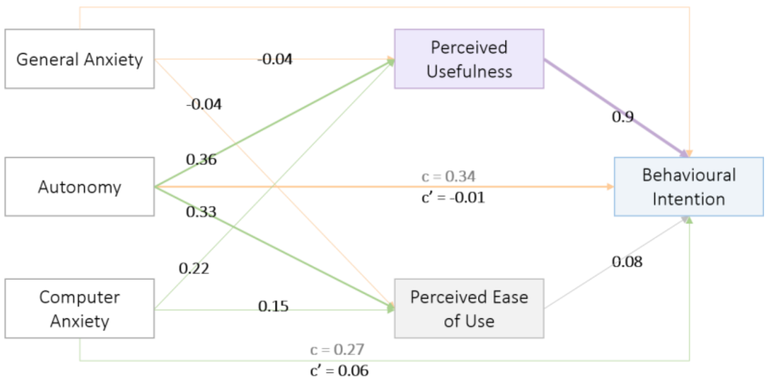

Figure S4. Mediation analysis for the ***individual differences***: *Autonomy* is the most influential factor, it equally impacts PU and PEOU. $R^2 = 0.87$ (p-value < 0.001)

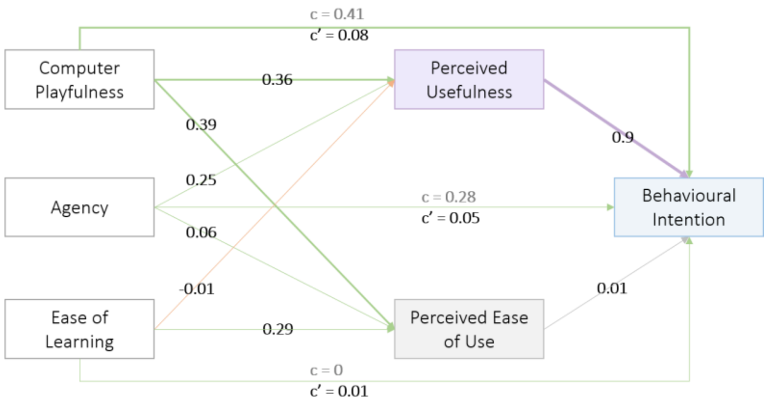

Figure S5. Mediation analysis for the ***facilitating conditions:*** *Computer playfulness* is the most influential factor, it equally impacts PU and PEOU. $R^2 = 0.87$ (p-value < 0.001)

3 CORRELATION ANALYSIS

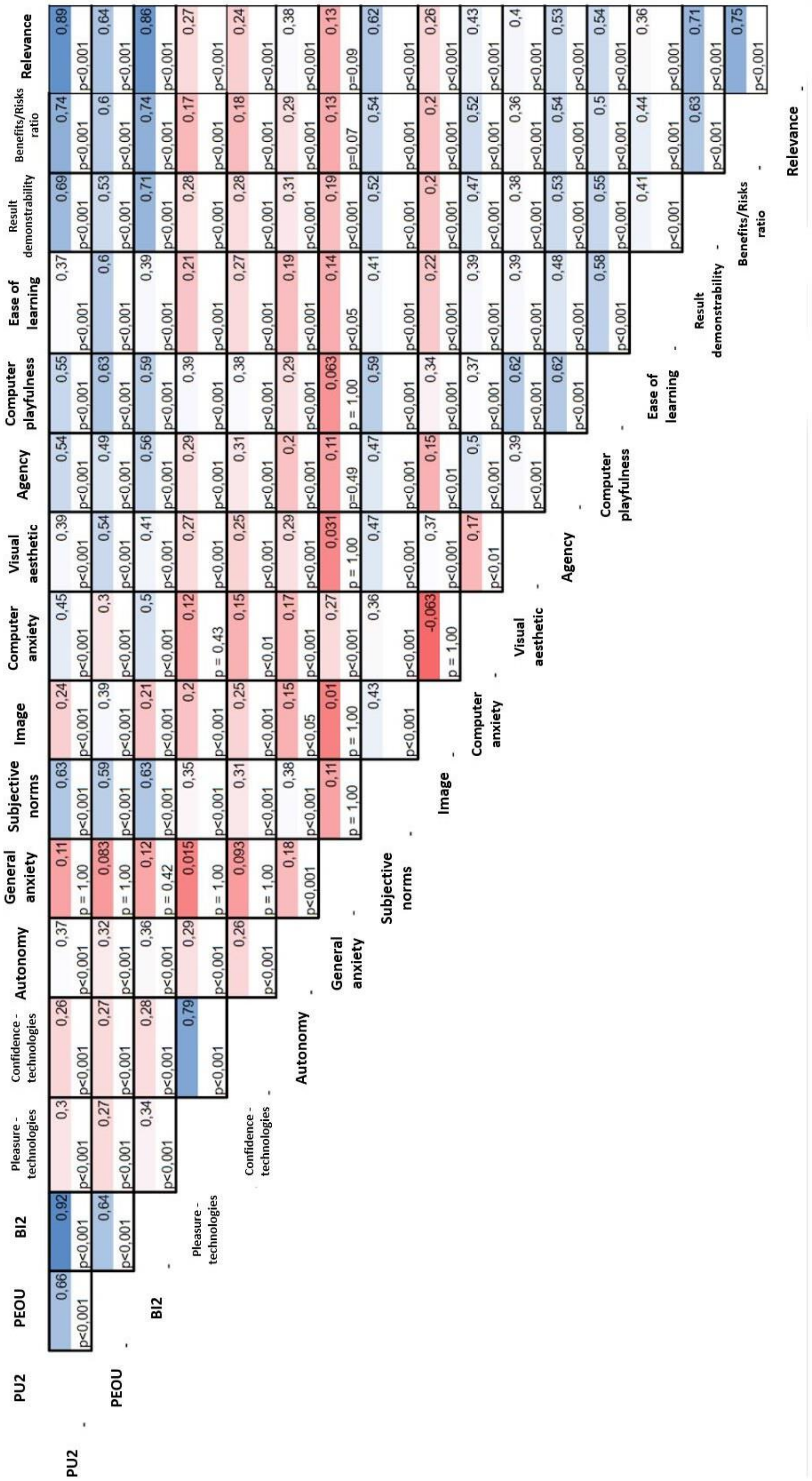

Figure S6. Correlation analysis (Spearman's coefficient) with all the quantitative factors of acceptability (excepted BI1 and PU1). *p-values adjusted using the Bonferroni method.*
